# Cross-sectional comparison of lower-limb muscle strength and contractile properties according to Parkinson’s disease and sarcopenia status

**DOI:** 10.3389/fmed.2026.1546672

**Published:** 2026-03-20

**Authors:** Sunghoon Shin, Yungon Lee

**Affiliations:** 1 Neuromuscular Control Laboratory, Yeungnam University, Gyeongsan, Gyungbuk, Republic of Korea; 2 Research Institute of Human Ecology, Yeungnam University, Gyeongsan, Gyungbuk, Republic of Korea

**Keywords:** ASM/height^2^ ratio, contractility, muscle strength, Parkinson’s disease, sarcopenia, tensiomyography

## Abstract

This cross-sectional study compares lower-limb isometric strength and tensiomyography-derived contractile properties between patients with Parkinson’s disease and age-matched controls across sarcopenia classifications, with predefined covariate control. However, despite the clinical relevance of muscle dysfunction in Parkinson’s disease and sarcopenia, TMG-derived assessments of muscle contractile properties remain limited in these populations. PD is a progressive neurodegenerative disorder marked by motor symptoms such as bradykinesia and rigidity, while sarcopenia is an age-related condition characterized by loss of muscle mass and strength. Both contribute to impaired mobility and increased fall risk, though their physiological signatures may differ. In this cross-sectional study, 60 older adults aged 58–85 years (20 with PD and 40 age-matched healthy controls; 39 males, 21 females) were assessed for isometric muscle strength across eight major lower limb movements using a handheld dynamometer, and for muscle contractile properties of four muscles—lateral gastrocnemius, medial gastrocnemius, soleus, and tibialis anterior—using tensiomyography (TMG). Compared to healthy controls, individuals with PD showed prolonged contraction time (Tc, the time between 10 and 90% of maximal contraction) and reduced maximal muscle displacement (Dm, the peak radial displacement of the muscle belly) in the lateral gastrocnemius (e.g., Tc↑: + 23.6%, Dm↓: -17.8%), suggesting neuromuscular impairment. Sarcopenic participants exhibited lower peak isometric strength in hip and knee extension tasks (e.g., -29.5% in hip extension, -26.2% in knee extension based on maximal voluntary contraction), reflecting task-specific deficits likely related to proximal muscle mass loss. While these results are based on cross-sectional data and should be interpreted cautiously, they suggest the presence of differing neuromuscular patterns in PD and sarcopenia. The findings offer preliminary insights into how PD and sarcopenia may differentially affect muscle strength and contractile characteristics, which could inform future research and clinical considerations regarding lower limb function.

## 1 Introduction

Parkinson’s disease (PD) is a progressive neurodegenerative disorder that primarily affects the motor system due to the degeneration of dopaminergic neurons in the substantia nigra (1–3). This neuronal loss disrupts motor control, leading to characteristic symptoms such as bradykinesia (slow movement), rigidity, tremors, and postural instability (4, 5). As the disease progresses, these motor impairments contribute to a significant decline in muscle strength and overall physical function, severely impacting patients’ quality of life (6). Participants with PD are generally categorized based on the Hoehn-Yahr scale, and symptoms typically presented asymmetrically, affecting one side more severely than the other. PD is one of the most prevalent neurodegenerative disorders globally, with its incidence rising due to the aging population (7). According to the World Health Organization, Parkinson’s disease currently affects millions of individuals worldwide (8). Recent global burden projections indicate that the number of people living with Parkinson’s disease is expected to more than double by 2050, largely driven by population aging and increased life expectancy (9).

PD induces peripheral muscular changes. Patients frequently exhibit muscle weakness, particularly in the lower extremities, which is often accompanied by muscle atrophy (10, 11). These changes impair their ability to perform daily tasks and contribute to a decline in physical performance. PD not only disrupts central motor control but also induces peripheral muscle changes. Histological and neuromuscular studies have shown that individuals with PD may exhibit an increased proportion of slow-twitch (Type I) muscle fibers—likely due to motor unit remodeling and chronic denervation–reinnervation processes—which are less efficient at generating force (12–15). Reduced physical activity further exacerbates muscle deterioration, creating a vicious cycle of muscle loss and functional decline (16, 17).

Sarcopenia, a condition characterized by the loss of skeletal muscle mass and function, has emerged as a major concern with the aging population (18). It significantly impacts mobility and increases the risk of falls, fractures, and disability (19). Sarcopenia is often diagnosed by measuring the appendicular skeletal muscle mass (ASM), particularly the ASM/height^2^ ratio, which is a key indicator of muscle health (20–22). Although no global consensus exists, both the European Working Group on Sarcopenia in Older People (EWGSOP) and the Asian Working Group for Sarcopenia (AWGS) recommend evaluating low muscle mass along with muscle strength and physical performance for diagnosis. Moreover, sarcopenia has been associated with several factors, including hormonal changes, inflammation, physical inactivity, and malnutrition, which contribute to muscle fiber degradation and the infiltration of fat and connective tissue into the muscle (20, 21).

Diagnostic criteria for Asian populations have also been adapted to account for different factors influencing sarcopenia (22). Sarcopenia affects not only elderly individuals but also patients with chronic diseases like PD, making them more susceptible to physical disability, falls, and further functional decline (23). As sarcopenia progresses, muscle fibers undergo structural and functional changes that decrease their contractile ability. A notable characteristic of sarcopenia is the reduction in fast-twitch fibers, which are responsible for generating high force contractions (24).

Tensiomyography (TMG) has been increasingly used to evaluate changes in muscle contractility in patients with sarcopenia by measuring parameters such as contraction time (Tc) and muscle displacement (Dm), which are useful for assessing muscle stiffness and fatigue resistance (25). Studies have shown that patients with sarcopenia exhibit longer Tc and reduced Dm, indicating decreased muscle elasticity and slower contraction speeds (26). These findings suggest that TMG could be an effective tool for diagnosing and monitoring sarcopenia in clinical settings.

Previous studies have highlighted the importance of isometric strength evaluation in assessing motor symptoms, functional impairments, and disease progression in patients with PD (23, 26, 27). Given the accuracy of maximal strength measurements, isometric testing provides reliable data to guide therapeutic interventions (28). Moreover, randomized controlled trials have shown that isometric strength training significantly improves both motor and non-motor symptoms in patients with PD, consequently enhancing motor control, balance, and overall function (29). Notably, isometric strength has been recognized as a reliable measure of muscle function in sarcopenia research, as it correlates strongly with muscle quality and neuromuscular function (11, 21). In older adults, reductions in isometric strength are closely associated with impaired physical performance, risk of disability, and loss of independence (21, 30). These findings highlight the crucial role of isometric strength evaluation in understanding and managing physical decline in both PD and sarcopenia.

Although previous studies have addressed PD and sarcopenia individually, fewer have investigated their combined effects. The following section consolidates this background to provide rationale for the present investigation. Recent evidence indicates that the coexistence of PD and sarcopenia may lead to compounded deficits in muscle strength, motor function, and neuromuscular health, particularly among older adults (7, 31, 32). Despite the growing number of studies on muscle strength and function among patients with PD, there exists a noticeable gap in research investigating how sarcopenia exacerbates these impairments (7, 8, 33). For instance, only a few studies have specifically examined how a reduction in ASM/height^2^ (kg/m^2^) among patients with PD affects motor function and physical performance (7, 11, 31, 33). Given the increased risk of sarcopenia in patients with PD due to decreased physical activity and progressive motor decline, investigating the combined effects of both conditions on muscle function is crucial (7).

Although PD and sarcopenia are distinct conditions, their coexistence in older adults may lead to compounding effects on physical performance and neuromuscular function. Recent studies have also reported overlapping symptoms and compounding risks when sarcopenia and PD co-occur, especially in older populations. Sarcopenia has been shown to be highly prevalent in individuals with PD, contributing to greater functional decline, fall risk, and physical disability in this population (7, 31).

Despite the growing body of independent research on Parkinson’s disease (PD) and sarcopenia, studies that thoroughly examine the interaction between these two conditions remain scarce. Moreover, while several investigations have addressed their co-existence and combined impact on muscle strength and characteristics, few have applied advanced diagnostic techniques to capture these effects comprehensively. Previous research has shown that individuals with PD exhibit a higher prevalence of sarcopenia and a greater frequency of falls compared with those without PD, underscoring the clinical significance of muscle deterioration in this population (7, 31).

Although tensiomyography (TMG) has been increasingly used to assess muscle contractile properties in older adults (34), its application to individuals with concurrent PD and sarcopenia is still limited. Addressing this gap, the present study employed TMG alongside conventional strength assessments to clarify the independent and interactive effects of PD and sarcopenia on lower-limb muscle strength and contractile properties. By investigating how these two conditions jointly influence muscle function, this study aimed to enhance the understanding of neuromuscular deterioration in PD and to provide a basis for future longitudinal studies exploring the development of sarcopenia in this population.

## 2 Materials and methods

### 2.1 Participants

A total of 60 participants were included in this cross-sectional study: 20 patients with Parkinson’s disease (PD) and 40 controls matched for age, sex, height, and weight (1:2 ratio). Patients with PD were recruited from Do Neurology Clinic (Daegu, Republic of Korea) and diagnosed by board-certified neurologists specializing in movement disorders, according to internationally accepted criteria (e.g., UK Brain Bank criteria). Only patients within Hoehn and Yahr stages 1–3, indicating mild to moderate disease severity, were included. Individuals with atypical parkinsonism, Hoehn and Yahr stage ≥ 4, or a history of surgical intervention (e.g., deep brain stimulation) were excluded. Control participants were recruited from the local community via public advertisements and were free of neurological or musculoskeletal disorders. Both male and female individuals were included in the control group, as shown in Table 1.

**TABLE 1:** Demographic and physical characteristics according to Parkinson’s disease status and sarcopenia status.

Comparisons	PD vs. non PD comparisons			Stratified by sarcopenia classification			
Parameters	Controls (*N* = 40)	Parkinson’s (*N* = 20)	*P*-value	Non-Sarcopenia group (*N* = 35)	Functional deficit group (*N* = 14)	Muscle loss group (*N* = 11)	*P*-value
Age (years)	70.58 (6.15)	71.65 (6.83)	0.541	69.66 (6.20)	72.43 (6.56)	73.09 (6.07)	0.178
Height (cm)	164.77 (6.47)	165.09 (8.10)	0.870	165.15 (6.90)	167.28 (6.21)	160.92 (7.06)	0.071
Weight (kg)	65.26 (8.23)	65.55 (7.67)	0.897	67.18 (7.52)	67.38 (5.16)	56.98 (7.39)	< 0.001***
Sex (male, n)	26	13	0.49	21	11	7	0.47
Total subjects (Parkinson, n)	N.A	N.A	N.A	35(10)	14(5)	11(5)	0.20
ASM (kg)	19.44 (3.34)	19.99 (3.84)	0.568	20.24 (3.36)	20.47 (2.76)	16.55 (3.32)	0.004**
Percent body fat (%)	28.48 (6.07)	26.69 (8.97)	0.364	28.12 (7.51)	26.91 (6.32)	28.34 (7.45)	0.846
Fat-free mass (kg)	46.60 (6.65)	48.03 (7.94)	0.466	48.18 (6.83)	49.22 (5.36)	40.81 (6.72)	0.003**
Skeletal muscle mass (kg)	25.75 (7.35)	26.28 (4.80)	0.671	26.46 (4.16)	27.64 (3.98)	22.04 (4.08)	0.003**
BMI (kg/m^2^)	23.96 (2.60)	24.11 (2.84)	0.847	24.61 ((2.74)	24.12 (2.00)	21.98 (2.27)	0.014*
WHR	0.90 (0.04)	0.87 (0.05)	0.021*	0.90 (0.04)	0.89 (0.05)	0.88 (0.04)	0.768
No of falls in previous year	0	0.6 (1.39)	0.069	0.06 (0.23)	0.64 (1.64)	0.09 (0.30)	0.076
Hoehn and Yahr stage	0	1.7	< 0.001***	1.11 (1.16)	0.36 (0.81)	0.57 (0.89)	0.034*

Data are mean (SD) unless otherwise indicated. Statistical analyses: For PD vs. non-PD comparisons, continuous variables were compared using independent-samples t tests and categorical variables using χ^2^ tests. For sarcopenia classification (three groups), continuous variables were compared using one-way ANOVA (with Tukey’s HSD *post hoc* when applicable) and categorical variables using χ^2^ tests. Normality was screened with the Shapiro–Wilk test. Significance set at *p* < 0.05. *p < 0.05; **p < 0.01; ***p < 0.001. ASM, appendicular skeletal muscle mass; WHR, waist-to-hip ratio.

The 1:2 PD-to-control ratio was adopted to improve statistical power within the reference group, particularly in light of recruitment constraints commonly faced in PD research (35, 36). This design strategy has also been shown to enhance the reliability and precision of between-group comparisons (37, 38).

**FIGURE 1:**
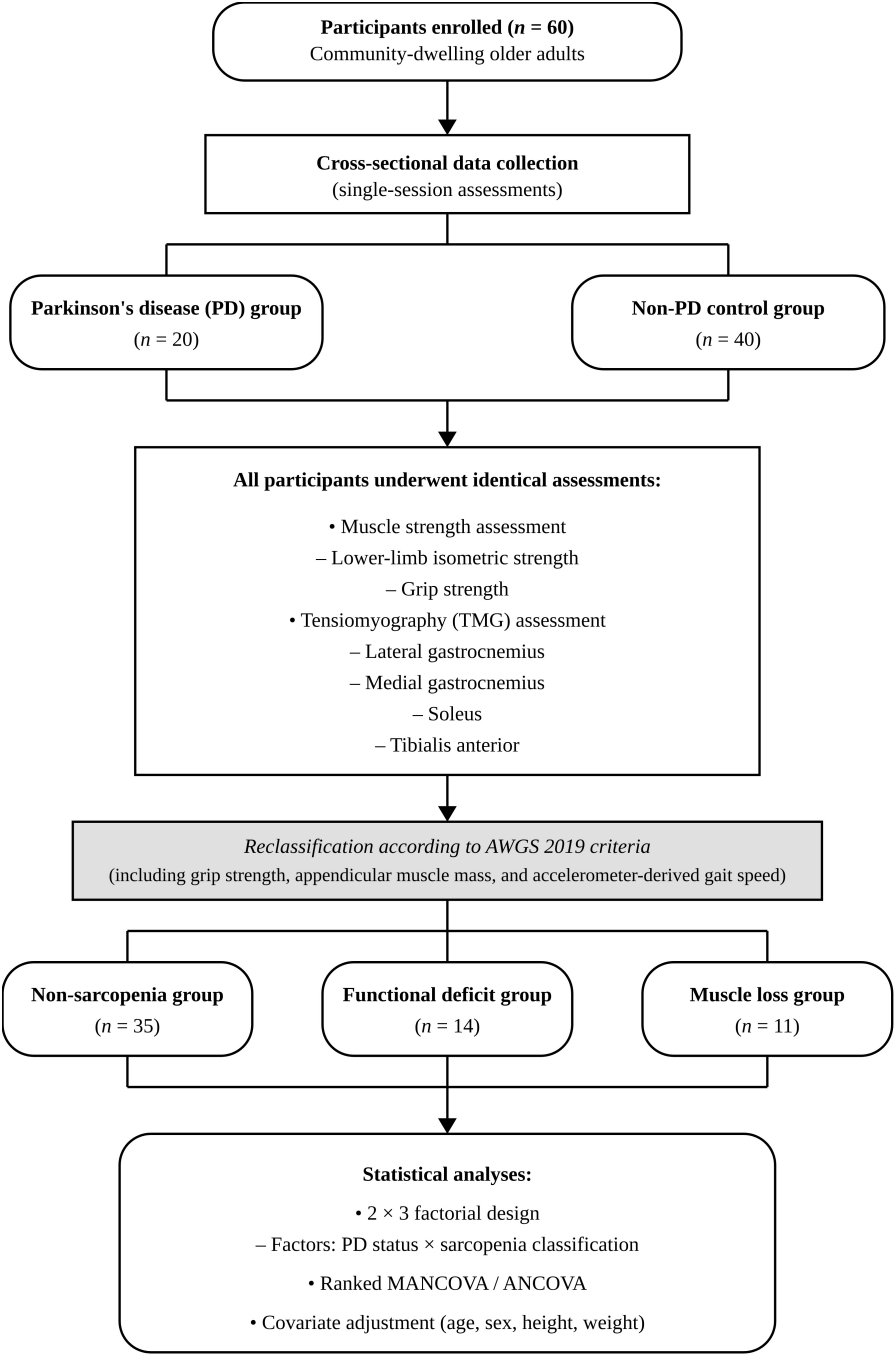
Study flow diagram. Flowchart illustrating participant recruitment, screening, and assessment procedures. Patients with Parkinson’s disease (PD) were referred by board-certified neurologists based on Movement Disorder Society (MDS) diagnostic criteria, and age-matched healthy controls were recruited from Yeungnam University. The final analysis included 20 participants with PD and 40 controls who completed clinical evaluation, body composition assessment, muscle strength testing, tensiomyography (TMG), and the 6-min walk test (6MWT).

PD severity was assessed using the Hoehn and Yahr scale and the Unified PD Rating Scale (UPDRS). Most patients were receiving standard pharmacological treatment, including levodopa-based therapy (e.g., levodopa/carbidopa or levodopa/benserazide) and dopamine agonists (e.g., pramipexole). In several cases, adjunct medications such as MAO-B inhibitors (rasagiline, selegiline), amantadine, benzodiazepines, or sleep aids were also prescribed. One participant was not taking any medications at the time of testing. A detailed list of medications for each participant with PD is presented in Supplementary Table 1. The overall recruitment, screening, and assessment flow is illustrated in Figure 1.

All participants with PD had a diagnosis duration of at least 1 year (range: 1.0–9.3 years; mean ± SD: 3.9 ± 2.1 years). Disease severity and functional status were evaluated using standardized clinical rating scales, including the Hoehn and Yahr scale, a motor function scale, and the Activities of Daily Living (ADL) scale. The Hoehn-Yahr scale was applied to classify disease stage from unilateral symptoms (stage 1) to severe bilateral disability (stage 5) (39). A motor function scale was used to assess movement impairments, and the ADL scale was used to evaluate patients’ ability to maintain independence.

Although the UPDRS is widely used in PD research, it was not uniformly available across all participants due to documentation variability in routine clinical care. In outpatient practice, physicians often prioritize time-efficient and validated assessments such as the Hoehn-Yahr and ADL scales. Nevertheless, disease severity was comprehensively assessed using the Hoehn-Yahr stage, a motor function scale, and an ADL scale. These measures, applied consistently across all subjects, provided a reliable and clinically valid basis for staging and analysis.

To explore the independent and combined effects of PD and sarcopenia, participants were further classified into three sarcopenia-related categories based on the Asian Working Group for Sarcopenia (AWGS) 2019 criteria: (1) non-sarcopenia group, (2) functional decline group (reduced muscle strength and/or physical performance with preserved muscle mass), and (3) muscle loss group (low appendicular skeletal muscle mass consistent with sarcopenia diagnosis). The classification criteria and grouping procedures—based on grip strength, 6-minute walking speed, and ASM/height^2^—are described in detail in section 2.5.1 Sample Characterization and Descriptive Statistics. Although sarcopenia is commonly identified using appendicular skeletal muscle mass (ASM), there is currently no universally accepted global definition of sarcopenia. Current consensus groups such as the EWGSOP2 and AWGS recommend a composite diagnostic approach that incorporates low muscle mass, reduced strength, and impaired physical performance. Given the regional relevance and population characteristics, the AWGS 2019 criteria were applied in this study.

Individuals were selected based on their ability to walk independently and their willingness to engage in physical activity, with exclusions for those with neuromuscular diseases, artificial joints, or metal implants. The specific physical characteristics of the subjects are summarized in Table 1. After body composition assessments, all participants underwent tests for muscle contraction characteristics and strength. All participants signed consent forms before participation, and this study was approved by the Institutional Review Board (IRB-2018-09-001-007).

### 2.2 Body composition assessment

The ASM/height^2^ ratio was derived using bioelectrical impedance analysis (InBody 370S; InBody Co., Ltd., Seoul, Korea), conducted in a standing position following the manufacturer’s protocol, to evaluate muscle mass relative to body size (40).

### 2.3 Muscle strength and functional assessment

Muscle strength of both upper and lower limbs was evaluated using standardized isometric testing protocols. To minimize fatigue and potential order effects, the sequence of isometric strength tasks was randomized across participants.

For the upper limbs, grip and pinch strength were assessed as complementary indicators of overall muscular function, in accordance with the Asian Working Group for Sarcopenia (AWGS 2019) diagnostic criteria, which recognize hand strength as a validated proxy for general strength decline. Grip and pinch strength of the dominant hand were measured using a JAMAR Hydraulic Hand Dynamometer (Sammons Preston, Bolingbrook, IL, United States) and a Jamar mechanical pinch gauge (Sammons Preston, Bolingbrook, IL, United States), respectively. Participants were seated upright with the elbow flexed at 90°, the forearm in a neutral position, and the wrist positioned between 0° and 30° extension. Each test was performed three times with 30-s rest intervals, and the highest value was used for analysis.

**FIGURE 2:**
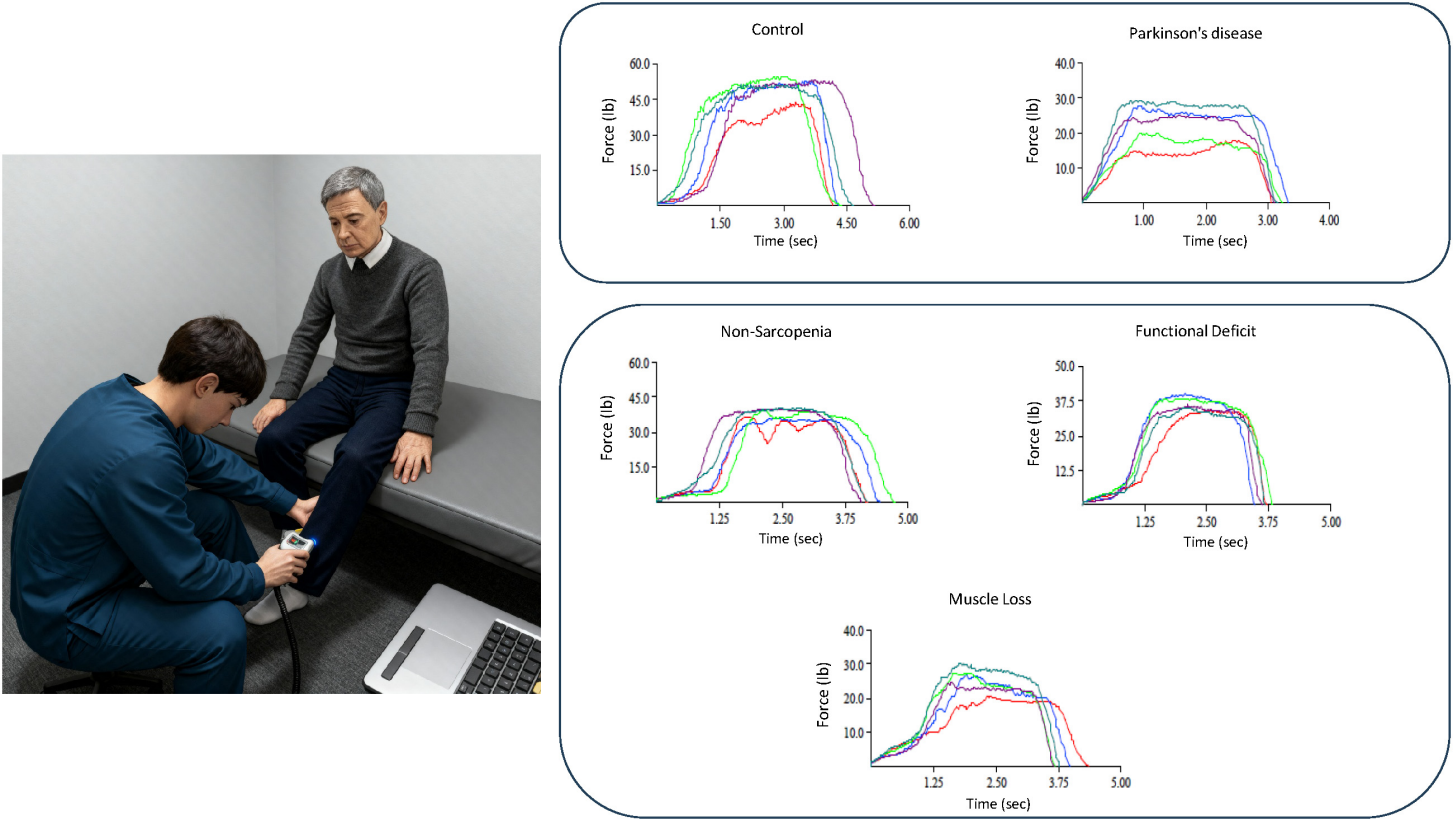
Representative muscle strength assessment postures. The figure on muscle strength presents data from a healthy control, a patient with Parkinson’s disease, and individuals classified as non-sarcopenic, functionally impaired, or exhibiting muscle loss. Lower limb muscle strength was assessed using a handheld isometric dynamometer, illustrating individual-level characteristics of knee extension strength.

For the lower limbs, a handheld isometric dynamometer (MicroFET; Hoggan Health Industries, West Jordan, UT, United States) was used to measure maximal voluntary contraction (MVC) forces during hip flexion, hip extension, hip abduction, knee flexion, knee extension, ankle plantarflexion, and dorsiflexion. These joint-specific assessments were selected to capture differential neuromuscular involvement associated with Parkinson’s disease (PD) and sarcopenia, particularly in relation to balance, gait, and mobility. Figure 2 illustrates the representative testing setup for one of these movements (knee extension), along with sample force-time curves from each participant group.

Each participant performed five MVC trials for each isometric task, and the force–time traces were color-coded (blue, red, brown, green, and black, respectively) to visualize trial consistency and maximal force determination. The highest MVC value among the five trials was used for analysis. Detailed descriptions of participant positioning, joint angles, and dynamometer placement are provided in Supplementary Table 2. All measurements adhered to the MicroFET standardized visual guides (shown in Supplementary Figure 1), which specified precise positioning, body stabilization, and joint alignment for each test, ensuring consistent reproduction of postures across participants and trials. These handheld dynamometer assessments have well-documented intra- and inter-rater reliability (ICC = 0.85–0.98) when performed by trained professionals. Given the investigator’s certification and adherence to standardized procedures, the collected muscle strength data were considered highly reliable with minimal measurement error.

All measurements were conducted at the Neuromuscular Control Laboratory, Yeungnam University, in a temperature-controlled environment (18–24°C) with quiet ambient conditions. Participants were instructed to abstain from alcohol, caffeine, and strenuous exercise for 24 h prior to testing. Upon arrival, participants rested for at least 20 min before the assessments, and all provided written informed consent. Equipment calibration was performed according to manufacturer instructions prior to testing.

Functional performance assessments, including the 6-min walk test (6MWT) used for sarcopenia classification, were conducted at the Cheonma Indoor Gymnasium, Yeungnam University, using a flat 30-m indoor track to ensure standardized conditions. Corner deceleration phases were automatically excluded using the GaitUp software’s built-in processing algorithm (41).

### 2.4 TMG for contractile muscle properties assessment

This study used a cross-sectional design with TMG measurements collected at a single time point, rather than through serial or longitudinal assessments. However, within each measurement session, the electrical stimulation intensity was progressively increased to identify the stimulus that elicited the maximal muscle response, as described below.

TMG measurements were conducted at rest, with the sensor placed over the anatomical belly of the lateral gastrocnemius (GL), medial gastrocnemius (GM), soleus (SO), and tibialis anterior (TA) muscles using established anatomical landmarks to evaluate the contractile properties of the same lower limb muscles. Using the TMG S1 device (TMG-BMC d.o.o., Ljubljana, Slovenia), non-invasive assessments were conducted of key muscle contractile properties, such as Tc, Dm, delay time (Td), sustained time (Ts), relaxation time (Tr), and contraction velocity (Vc).

**FIGURE 3:**
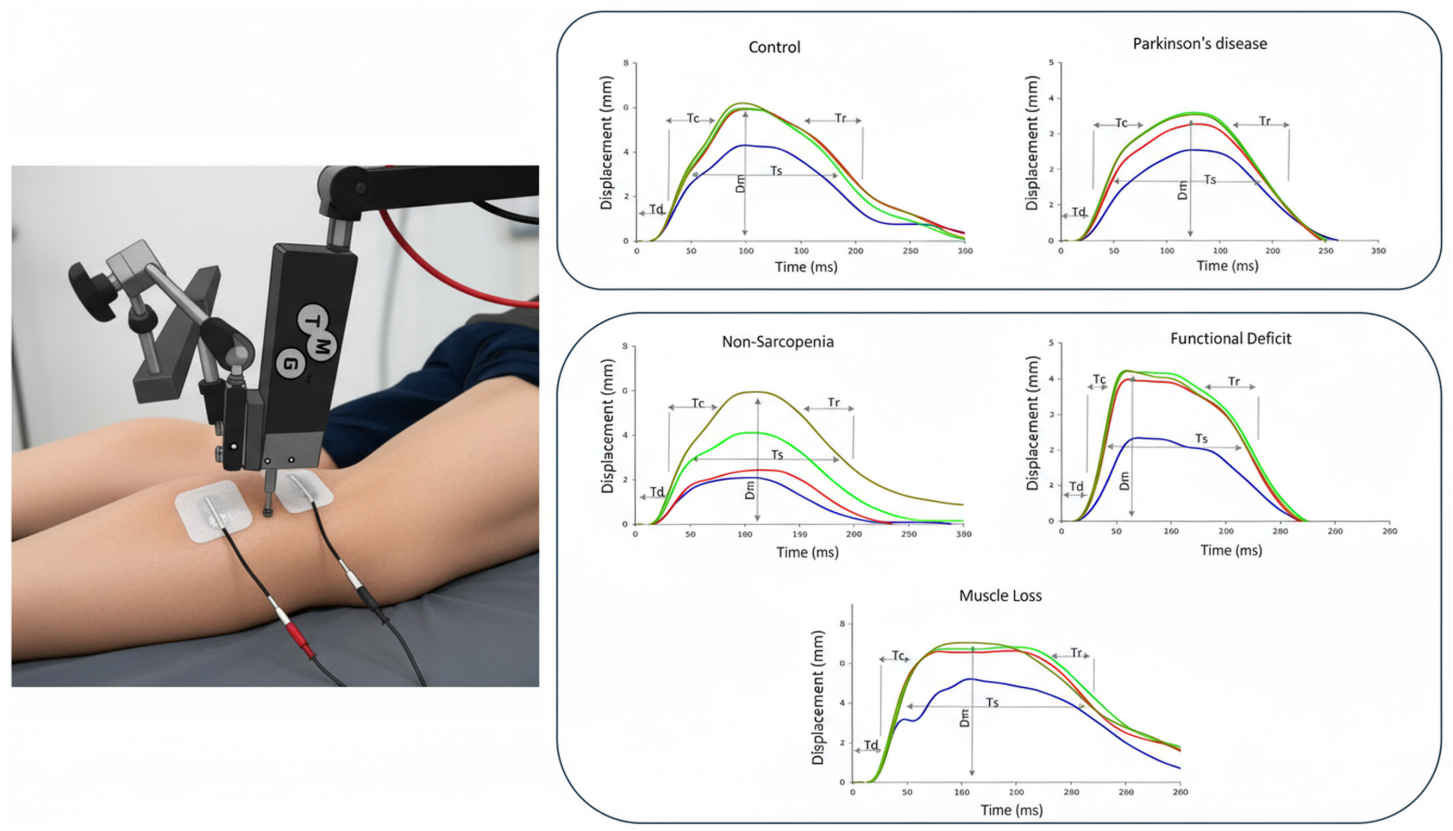
Representative muscle contractile property profiles. The figure on muscle contractile properties presents data from a healthy control, a patient with Parkinson’s disease, and individuals classified as non-sarcopenic, functionally impaired, or exhibiting muscle loss. Muscle contractile properties were assessed in the lower limb using a TMG device. The extracted parameters included contraction time (Tc), maximal displacement (Dm), delay time (Td), sustained time (Ts), and relaxation time (Tr). The figure illustrates individual-level characteristics of lateral gastrocnemius (GL) contractile properties.

Gel electrodes were attached to the muscle belly, and electrical stimulation with a 1-ms pulse duration was delivered at progressively increasing intensities in 20 mA increments, starting from 40 mA and increasing up to a maximum of 100 mA, until the maximum radial displacement was achieved. The stimulus that elicited the maximal muscle response was identified and recorded. For each muscle, four contraction responses were collected under these stepwise stimulation conditions, ensuring consistency in the identification of the maximal contractile response. TMG displacement–time curves were recorded at four stimulation intensities (40, 60, 80, and 100 mA), corresponding to the color-coded traces shown in Figure 3 (blue, red, green, and brown, respectively).

To quantify the muscle contractile response, specific parameters were analyzed (27, 42):

- Td: The time between the electrical impulse and 10% of the contraction. This parameter was specifically developed to provide a consistent and objective definition of the onset of muscle response, minimizing subjective interpretation.
- Tc: The time between 10 and 90% of the contraction, which reflects the muscle’s contractile speed. Tc is derived based on the predefined Td, ensuring that the measurement of contraction onset is standardized.
- Ts: The time between 50% of the contraction and 50% of the relaxation. This measure helps in understanding the duration of tension maintenance, which is a critical factor in muscle function assessment.
- Tr (relaxation time): The time between 90 and 50% of the relaxation phase, serving as a measure of how quickly the muscle returns to its resting state after contraction. This parameter is essential for evaluating muscle fatigue resistance and recovery characteristics.
- Dm: The peak muscle displacement during contraction, which has been widely used as an indicator of muscle stiffness and elasticity.
- Vc: Contraction velocity (Dm/Tc)

**FIGURE 4:**
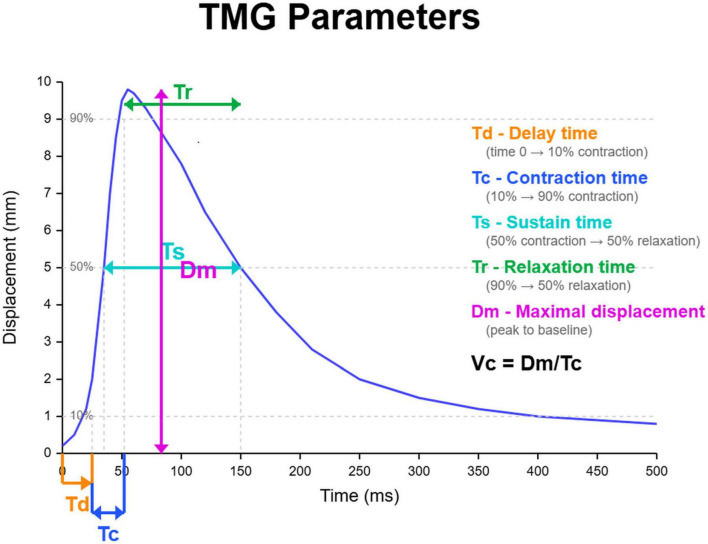
Illustrative schematic of TMG-derived parameters. Schematic displacement–time curve created for explanatory purposes to illustrate how tensiomyography (TMG) parameters are defined. Td, delay time (0–10% of Dm); Tc, contraction time (10–90% of Dm); Ts, sustain time (50% contraction → 50% relaxation); Tr, relaxation time (90 → 50% relaxation); Dm, maximal radial displacement; Vc, contraction velocity (Dm/Tc).

The relationships among these parameters and their temporal sequence are illustrated in Figure 4. These parameters (Td, Ts, Tr) were not arbitrarily chosen but were experimentally determined as the optimal points for defining contraction onset, maintenance, and relaxation. Td is critical in determining Tc, as it establishes a consistent baseline from which the muscle’s contraction speed is measured. Similarly, Ts and Tr help in interpreting Dm, ensuring that maximum displacement measurements are not influenced by transient or partial muscle activations. These parameters provide necessary standardization, allowing Tc and Dm to be compared reliably across different individuals and conditions.

Recent studies have also examined Vc, which represents the change in Dm over time between 10 and 90% of the contraction (26, 43). Given its relevance, Vc was included in this study. For example, Dm serves as an indicator of muscle stiffness, while Vc reflects contraction velocity. Participants were positioned supine for quadriceps assessments and prone for hamstring evaluations, with foam cushions used to support the required joint angles. Contractile muscle properties were evaluated for four key lower limb muscles: the GL, GM, SO, and TA (Figure 3).

To ensure consistency in measurement posture, TMG assessments were conducted in standardized positions based on muscle anatomy: participants were positioned supine for GL, GM, and TA, and prone for SO. Foam cushions—uniformly shaped and officially provided by the TMG equipment manufacturer—were used to precisely support joint angles and reduce postural variability. Furthermore, the exercise specialist who performed all measurements directly participated in the official expert training program provided by the TMG manufacturer, ensuring strict adherence to standardized procedures and enhancing the credibility of the collected data.

### 2.5 Statistical methods

#### 2.5.1 Sample characterization and descriptive statistics

The normality of the data was assessed using the Shapiro–Wilk test. Participants were initially divided into two primary groups based on Parkinson’s disease (PD) status: a PD group and a non-PD control group. Subsequently, in accordance with the Asian Working Group for Sarcopenia (AWGS) 2019 criteria, all participants were further classified into three sarcopenia-related subgroups: non-sarcopenic, functional decline, and muscle loss. These classifications were determined using grip strength, 6-min walk speed, and ASM/height^2^ ratio (22). Notably, none of the participants simultaneously met the criteria for both low physical function and low muscle mass. Therefore, the sarcopenia classification in this study reflects partial fulfillment of the AWGS 2019 definition (i.e., individuals met either the functional decline or low muscle mass criterion, but not both). Based on this classification structure, a 2 × 3 factorial design was applied, treating PD status (PD vs. control) and sarcopenia classification (non-sarcopenic, functional decline, muscle loss) as two independent factors in multivariate analyses. This design allowed for the examination of both the main effects and potential interaction effects of these factors on muscle strength and contractile properties.

The non-sarcopenia group consisted of participants who exceeded the thresholds for all three indicators: grip strength, 6-min walking speed, and ASM/height^2^ ratio. Specifically, the thresholds used were as follows:

- Grip Strength: ≥ 26 kg for men and ≥ 18 kg for women (based on the AWGS 2019 criteria).
- 6-min Walking Speed: ≥ 1.0 m/s, indicating adequate physical performance.
- ASM/height^2^: ≥ 7.0 kg/m^2^ for men and ≥ 5.7 kg/m^2^ for women, which is the established threshold for diagnosing sarcopenia.

In contrast, the functional decline group comprised individuals who scored below the thresholds for both grip strength and 6-min walking speed. Finally, participants whose ASM/height^2^ ratio was below the established threshold for sarcopenia were categorized into the muscle loss group.

#### 2.5.2 Homogeneity of covariance matrices

To ensure the appropriateness of subsequent multivariate analyses, the homogeneity of the covariance matrices for each dependent variable was tested using Box’s M test. The preliminary analysis indicated that the assumption of homogeneity was not met, which prompted us to perform a series of two-way ranked multivariate analyses of covariance (MANCOVA).

#### 2.5.3 Two-way ranked MANCOVA for muscle strength

The first analysis aimed to explore the interaction effects between PD status (PD vs. non-PD) and sarcopenia status (non-sarcopenia, functional decline, muscle loss) on various muscle strength measures. The independent variables were PD status and sarcopenia status, whereas the dependent variables included a comprehensive range of muscle strength assessments that captured the functional capacities of both the upper and lower body. These assessments included measurements of MVCs during hip flexion, hip extension, hip abduction, knee flexion, knee extension, ankle plantar flexion, and ankle dorsiflexion, grip strength, and pinch force.

#### 2.5.4 Two-way ranked MANCOVA for contractile muscle properties

The second analysis aimed to explore the interaction effects between PD status (PD vs. non-PD) and sarcopenia status (non-sarcopenia, functional decline, muscle loss) on various contractile muscle properties. Independent variables were the same as those in the previous MANCOVA. To examine these interactions, six separate two-way ranked MANCOVAs were conducted. Each MANCOVA analyzed a distinct contractile property (Td, Tc, Ts, Tr, Dm, and Vc) across the four assessed lower limb muscles (GL, GM, SO, and TA). This approach was used to retain the interpretive value of variable-specific findings.

#### 2.5.5 Covariates

Potential covariates, including age, height, weight, and sex, were incorporated into the analyses to control for their influence. Sex was included as a covariate because both male and female participants were enrolled, and prior evidence indicates that sex influences muscle strength and contractile behavior. After running multiple MANCOVAs with various potential covariates, sex was consistently identified as a significant covariate and was retained in the final model. In cases where interaction effects between PD status and sarcopenia status were detected, a two-way ranked analysis of covariance (ANCOVA) was performed. When no interaction effects were present, univariate analysis was conducted to assess the main effects of each independent variable on the dependent variables.

This approach ensured a thorough and robust analysis of the relationship between PD, sarcopenia, and muscle function, accounting for any potential confounding factors that might influence the results.

## 3 Results

To maintain consistency with the classification outlined in section 2.4.1 (Sample Characteristics), the sarcopenia-related categories analyzed in this section are defined as follows: (1) non-sarcopenia group, (2) functional decline group (participants with reduced muscle strength and/or performance but preserved muscle mass), and (3) muscle loss group (participants with confirmed low muscle mass consistent with sarcopenia diagnosis).

All muscle strength values were analyzed and reported as absolute values. Although baseline demographic comparisons (see Table 1) revealed significant differences in some anthropometric variables (e.g., WHR between PD and control groups; Weight and BMI across sarcopenia groups), these variables were evaluated as potential covariates, and sex was retained and statistically controlled in the final multivariate models (see section 2.5.5). Furthermore, muscle mass for individual body regions was not assessed; therefore, muscle strength could not be normalized to local muscle mass, and normalization was not performed. This approach (using absolute values with covariate adjustment) was thus deemed appropriate. To enhance interpretability and address the multidimensionality of the data, results are presented in two parts. First, multivariate outcomes derived from two-way ranked MANCOVA analyses are reported for isometric muscle strength and neuromuscular contractile properties. For a comprehensive overview, Supplementary Table 3 summarizes the full MANCOVA outputs, including Wilks’ Lambda values, degrees of freedom, F statistics, exact *p*-values, and partial eta-squared values. Second, *post hoc* univariate tests are used to identify specific between-group differences for each muscle or parameter. To avoid redundancy and improve readability, only statistically significant results (*p* < 0.05) are explicitly described in the text, while the full set of *Post Hoc* Ranked ANCOVAs is provided in Supplementary Table 4. Pairwise comparisons for isometric strength and TMG-derived contractility are presented in Supplementary Tables 5, 6, respectively.

Given that the assumption of homogeneity of covariance matrices was not met (Box’s M test, *p* < 0.001; see section 2.4.2), all multivariate analyses were performed using ranked data. This non-parametric approach was chosen to ensure robust inference across groups with potentially unequal covariance structures.

Table 2 presents univariate ANOVA comparisons for individual isometric forces or contractile parameters, which may not fully reflect the joint effects captured in the multivariate models. While knee extension strength in PD participants deviated from the overall trend, the multivariate model confirmed a broader pattern of strength reduction across multiple isometric force tasks, reinforcing the utility of MANCOVA in analyzing complex, multidimensional data structures. Although ranked data were used in the multivariate analyses, raw means and standard errors are presented in Table 2 to offer descriptive insight into group-level differences, facilitating intuitive interpretation of the underlying data patterns.

**TABLE 2:** Means and standard errors for all of and contractile muscle properties according to Parkinson’s disease status and sarcopenia status.

Comparisons	PD vs. non PD comparisons			Stratified by sarcopenia classification			
Parameters	Controls (*N* = 40)	Parkinson’s (*N* = 20)	*P*-value^a^	Non-sarcopenia group (*N* = 35)	Functional deficit group (*N* = 14)	Muscle loss group (*N* = 11)	*P*-value^b^
Grip strength	29.55 (5.97)	27.93 (9.19)	0.415	30.99 (7.03)	25.88 (6.56)	26.69 (6.76)	0.036*
Pinch force	6.20 (1.72)	6.64 (1.65)	0.350	6.50 (1.64)	6.85 (1.70)	5.21 (1.53)	0.038*
Hip flexion	40.56 (9.37)	45.80 (14.38)	0.095	44.30 (11.96)	40.91 (11.25)	37.72 (9.00)	0.221
Hip extension	35.96 (12.07)	32.88 (12.55)	0.362	38.00 (11.83)	28.65 (9.77)	33.19 (13.76)	0.044*
Hip joint _abduction	30.85 (7.87)	25.99 (8.66)	0.034*	30.65 (8.78)	27.17 (7.14)	27.32 (8.36)	0.305
Knee flexion	22.29 (8.37)	18.95 (8.37)	0.125	22.77 (8.14)	19.22 (7.24)	18.61 (7.59)	0.185
Knee extension	46.75 (13.07)	55.83 (19.26)	0.035*	53.06 (16.38)	43.93 (13.87)	46.77 (14.94)	0.150
Ankle plantar flexion	43.60 (10.03)	35.43 (11.03)	0.006**	43.73 (9.98)	38.06 (10.61)	35.38 (12.44)	0.046*
Ankle dorsiflexion	35.14 (8.73)	35.07 (9.78)	0.975	37.48 (8.50)	31.75 (9.83)	31.89 (7.83)	0.053
GL Td	24.78 (3.87)	22.43 (3.44)	0.025*	24.34 (3.88)	24.02 (4.21)	22.85 (3.47)	0.546
GL Tc	42.40 (18.34)	27.13 (13.44)	0.002**	36.80 (17.62)	34.97 (17.46)	41.89 (21.94)	0.630
GL Ts	219.97 (71.79)	211.32 (88.61)	0.686	214.63 (74.54)	209.68 (72.82)	234.29 (94.92)	0.708
GL Tr	63.11 (35.54)	45.84 (25.75)	0.058	58.63 (32.46)	45.45 (20.66)	68.46 (45.91)	0.221
GL Dm	4.50 (2.96)	3.04 (2.21)	0.056	4.11 (2.51)	3.15 (2.58)	4.79 (3.79)	0.333
GL Vc	0.11 (0.07)	0.11 (0.07)	0.905	0.12 (0.08)	0.09 (0.07)	0.10 (0.07)	0.639
GM Td	26.07 (6.13)	21.98 (3.83)	0.009**	24.48 (4.73)	24.88 (7.02)	25.24 (7.50)	0.925
GM Tc	32.53 (13.62)	21.77 (3.93)	< 0.001***	30.39 (15.01)	25.27 (7.94)	29.94 (12.40)	0.434
GM Ts	228.77 (121.96)	286.10 (143.46)	0.111	244.07 (127.83)	245.26 (85.59)	263.34 (189.97)	0.913
GM Tr	51.65 (33.60)	70.72 (58.50)	0.114	54.99 (34.37)	61.91 (50.44)	62.66 (63.16)	0.824
GM Dm	2.53 (1.42)	2.33 (1.14)	0.574	2.54 (1.40)	1.91 (0.93)	2.93 (1.35)	0.142
GM Vc	0.08 (0.04)	0.11 (0.04)	0.075	0.09 (0.04)	0.07 (0.03)	0.10 (0.05)	0.328
SO Td	26.43 (5.99)	23.08 (5.94)	0.045	24.66 (5.83)	23.95 (4.25)	29.12 (7.94)	0.068
SO Tc	45.10 (14.98)	36.14 (21.90)	0.067	40.19 (16.36)	40.54 (20.64)	50.23 (18.46)	0.254
SO Ts	233.31 (177.44)	262.54 (114.93)	0.507	234.17 (139.21)	287.48 (231.60)	214.78 (97.44)	0.468
SO Tr	47.41 (19.32)	82.77 (73.24)	0.046*	52.98 (21.68)	61.67 (55.05)	75.84 (85.59)	0.376
SO Dm	2.58 (1.69)	1.79 (1.25)	0.073	2.28 (1.32)	2.34 (2.08)	2.38 (1.82)	0.983
SO Vc	0.07 0.06)	0.06 (0.03)	0.613	0.07 (0.07)	0.06 (0.03)	0.05 (0.04)	0.738
TA Td	24.35 (10.62)	53.17 (36.65)	0.002**	33.91 (21.71)	37.46 (34.71)	29.63 (29.86)	0.768
TA Tc	18.37 (8.69)	26.10 (15.27)	0.046*	20.43 (9.28)	23.64 (17.49)	19.15 (10.57)	0.598
TA Ts	252.51 (162.34)	245.07 (66.32)	0.845	249.11 (142.45)	241.45 (98.26)	263.89 (171.34)	0.992
TA Tr	49.59 (62.87)	31.67 (43.27)	0.257	43.20 (64.05)	45.05 (53.78)	43.15 (41.08)	0.995
TA Dm	1.25 (1.03)	0.77 (0.37)	0.012**	1.02 (0.87)	1.18 (1.08)	1.19 (0.77)	0.792
TA Vc	0.07 (0.05)	0.03 (0.01)	< 0.001***	0.05 (0.04)	0.05 (0.05)	0.06 (0.04)	0.703

Muscle strength variables indicate Maximal voluntary contraction (MVC) force (kg), and tensiomyography (TMG) parameters are reported in milliseconds (ms) and displacement (mm), as appropriate. ^a^ANOVA main effect for PD status. ^b^ANOVA main effect for sarcopenia status. MVC, maximum voluntary contraction; Td, delay time; Tc, contraction time; Ts, sustain time; Tr, relaxation time; Dm, maximal Displacement; GL, lateral gastrocnemius; GM, medial gastrocnemius; SO, soleus; TA, tibialis anterior. Significance set at *p* < 0.05. *p < 0.05; **p < 0.01; ***p < 0.001.

### 3.1 Isometric muscle strength according to PD and sarcopenia status

A two-way ranked multivariate analysis of covariance (MANCOVA) was conducted to examine the effects of PD status (PD vs. non-PD) and sarcopenia status (non-sarcopenia, functional decline, muscle loss) on isometric muscle strength, as measured by MVC forces across isometric force tasks, while controlling for sex.

Multivariate analysis revealed a non-significant interaction effect between PD and sarcopenia status on MVC force during isometric muscle contraction [Wilks’ Λ = 0.691, *F*(18, 90) = 1.014, *p* = 0.453, η^2^ = 0.169]. However, significant main effects were observed for PD status [Wilks’ Λ = 0.598, *F*(9, 45) = 3.358, *p* = 0.003, η^2^ = 0.402], sarcopenia status [Wilks’ Λ = 0.430, *F*(18, 90) = 2.625, *p* = 0.001, η^2^ = 0.344], and sex [Wilks’ Λ = 0.347, *F*(9, 45) = 9.427, *p* < 0.001, η^2^ = 0.653]. Collectively, these results indicate that PD was associated with selective reductions in isometric MVC force while sarcopenia was linked to more generalized force deficits across multiple isometric force tasks involving both upper and lower limbs.

*Post hoc* univariate analyses revealed significant group differences in specific isometric force outputs. Compared to the non-PD group, the PD group exhibited significantly lower MVC forces in hip abduction [*F*(1, 50) = 4.72, *p* = 0.035] and ankle plantar flexion [*F*(1, 50) = 9.87, *p* = 0.003], but unexpectedly higher force in knee extension [*F*(1, 50) = 4.31, *p* = 0.043].

Regarding sarcopenia status, participants in the functional decline and muscle loss groups showed significantly lower forces in grip strength [*F*(2, 50) = 10.83, *p* < 0.001], pinch [*F*(2, 50) = 5.29, *p* = 0.008], hip extension [*F*(2, 50) = 7.41, *p* = 0.002], hip flexion [*F*(2, 50) = 6.14, *p* = 0.004], knee extension [*F*(2, 50) = 5.01, *p* = 0.010], and ankle dorsiflexion [*F*(2, 50) = 8.63, *p* = 0.001] compared to the non-sarcopenia group.

These findings indicate that PD may selectively affect specific lower limb muscles, whereas sarcopenia more broadly compromises force production across multiple isometric force tasks. The absence of a significant interaction effect suggests that the two conditions independently contribute to strength impairments.

### 3.2 Contractile muscle properties according to PD and sarcopenia status

To evaluate differences in neuromuscular contractile properties, a series of two-way ranked MANCOVAs were conducted for each TMG parameters (Td, Tc, Ts, Tr, Dm, and Vc) across four lower limb muscles: the lateral gastrocnemius (GL), medial gastrocnemius (GM), soleus (SO), and tibialis anterior (TA), controlling for sex.

Multivariate analysis revealed a significant interaction effect between PD and sarcopenia status only for Ts (sustained contraction time) [Wilks’ Λ = 0.719, *F*(8, 100) = 2.238, *p* = 0.031, η^2^ = 0.152]. However, *post hoc* analyses revealed no significant differences in Ts across sarcopenia classification groups when analyzed separately within the PD and control groups. Significant main effects of PD status were also observed for Td [Wilks’ Λ = 0.706, *F*(4, 50) = 5.209, *p* = 0.001, η^2^ = 0.294], Tc [Wilks’ Λ = 0.638, *F*(4, 50) = 7.104, *p* < 0.001, η^2^ = 0.362], Ts [Wilks’ Λ = 0.806, *F*(4, 50) = 3.008, *p* = 0.027, η^2^ = 0.194], Tr [Wilks’ Λ = 0.755, *F*(4, 50) = 4.046, *p* = 0.006, η^2^ = 0.245], and Vc [Wilks’ Λ = 0.769, *F*(4, 50) = 3.753, *p* = 0.010, η^2^ = 0.231]. In contrast, no significant main or interaction effects were found for Dm. Sarcopenia status did not yield significant main effects for any TMG parameter.

*Post hoc* univariate analyses also identified significant between-group differences in neuromuscular contractile parameters. The PD group showed significantly reduced Td in the lateral gastrocnemius [GL; *F*(1, 50) = 5.93, *p* = 0.024] and medial gastrocnemius [GM; *F*(1, 50) = 4.72, *p* = 0.035], as well as shorter Tc in the same muscles [GL: *F*(1, 50) = 11.52, *p* = 0.001; GM: *F*(1, 50) = 10.64, *p* = 0.001], compared to the non-PD group.

In contrast, the tibialis anterior (TA) showed prolonged Td and Tc in the PD group [Td: *F*(1, 50) = 7.92, *p* = 0.007; Tc: *F*(1, 50) = 5.43, *p* = 0.021], suggesting a muscle-specific disruption pattern.

Additionally, the PD group demonstrated higher Tr values in the soleus [SO; *F*(1, 50) = 8.48, *p* = 0.005) and lower Dm in the GL and SO [GL: *F*(1, 50) = 5.62, *p* = 0.024; SO: *F*(1, 50) = 5.71, *p* = 0.024), indicating increased muscle stiffness and slower recovery kinetics.

Sarcopenia status did not yield significant effects in any individual TMG parameter after *post hoc* correction, despite a multivariate interaction effect observed in Ts.

Overall, the results indicate that PD has a greater impact on neuromuscular contractile properties than sarcopenia, which appears to predominantly affect muscle strength.

## 4 Discussion

This study examined the independent and potential interactive effects of PD and sarcopenia on lower limb muscle strength and contractile properties using objective neuromechanical assessments. We hypothesized that each condition would independently impair muscle function, while also exploring the possibility of interaction.

Our findings partially supported this hypothesis, revealing distinct neuromechanical patterns for each condition without a significant interaction effect. Overall, the present findings highlight distinct yet partly overlapping neuromuscular characteristics between PD and sarcopenia.

First, our findings showed that Parkinson’s disease (PD) and sarcopenia had significant main effects on isometric muscle strength, whereas no interaction effect was observed between the two conditions. Among the nine MVC force assessments, PD participants demonstrated significantly reduced strength in most muscles. Notably, however, they exhibited relatively higher strength in knee extension compared to non-PD controls, which contrasts with the overall pattern. This deviation may reflect localized variations in muscle activation or functional preservation specific to certain tasks. Despite this exception, the multivariate analysis (MANCOVA) confirmed an overall trend of reduced strength in the PD group across all isometric force tasks. This supports the value of multivariate approaches in detecting group-level patterns while accommodating variable-specific anomalies.

These nine isometric strength assessments were selected to provide a comprehensive yet feasible representation of lower limb function, encompassing hip flexion and extension, knee flexion and extension, and ankle plantar and dorsiflexion. This selection aligns with previous studies assessing functional impairments in populations at risk of motor dysfunction, including older adults and individuals with PD or sarcopenia (44–46). In addition to the lower limb assessments directly related to gait and postural control, grip strength was measured as a surrogate marker of global muscle strength, consistent with its role in sarcopenia diagnostic criteria (AWGS 2019). Pinch strength was additionally assessed to complement grip strength measurements, providing further insights into fine motor function and precision grip capacity, which are often sensitive to neuromuscular impairments such as those observed in PD (47). This integrated approach allowed for a more comprehensive evaluation of musculoskeletal decline in aging and neurodegenerative populations (48).

The differential pattern of strength observed suggests that PD may selectively affect specific isometric force tasks. For instance, while the MVC forces of hip abduction and ankle plantar flexion were significantly lower in the PD group, knee extension strength was higher. This differential distribution of muscle strength supports previous reports that PD does not produce a uniform pattern of motor impairment but rather leads to muscle-specific deficits (49). For example, while hip abduction and plantar flexion strength were significantly reduced in the PD group, knee extension strength was unexpectedly higher. This imbalance cannot be attributed solely to global strength decline or measurement variability. Previous studies have shown that such selective impairments are characteristic of PD. Inkster et al. (49) demonstrated that muscular weakness in PD varies by limb and joint, indicating region-specific effects. David et al. (50) found that distal postural muscles, such as the plantar flexors, are more vulnerable than knee extensors, and Lima et al. (31) observed that hip abductors and plantar flexors show greater performance deficits in PD than other muscle groups. Collectively, these findings provide a neurophysiological basis for the muscle-specific strength distribution observed in our PD cohort. These observations are broadly in line with previous reports, although minor discrepancies across muscle groups may stem from methodological differences or sample variability.

Second, distinct neuromechanical alterations were identified through TMG, particularly among individuals with PD. These TMG-based indicators—such as elevated Tc and reduced Dm—reflect impairments in neuromuscular activation and muscle-tendon stiffness, offering physiological insights into disease-specific muscle dysfunction (26). In individuals with PD, such alterations suggest diminished motor unit recruitment efficiency and increased passive resistance, which may affect both movement initiation and coordination. Accordingly, rehabilitation strategies for PD should emphasize explosive strength training, neuromuscular coordination, and dynamic balance to improve motor responsiveness and functional mobility(29, 51).

In contrast, participants with sarcopenia demonstrated global reductions in isometric muscle strength, but TMG parameters remained largely unchanged. This pattern suggests that strength loss in sarcopenia may arise more from structural deterioration—such as reductions in muscle cross-sectional area or infiltration of fat and connective tissue—than from intrinsic changes in contractile dynamics (20). A previous study has reported that in mild-to-moderate sarcopenia, electrical responsiveness may remain intact even when force-generating capacity declines (34). Therefore, resistance training programs focusing on hypertrophy and restoration of contractile mass, particularly in proximal muscles such as the quadriceps and gluteals, may be effective for mitigating sarcopenic weakness (48).

Moreover, although our study did not observe statistically significant differences in TMG variables across sarcopenia classification groups, this may be due to the relatively moderate severity of muscle loss in the cohort. Previous studies involving older adults without formally diagnosed sarcopenia have shown that prolonged Tc and reduced Dm are associated with poorer balance and mobility performance, suggesting early neuromechanical deficits (52, 53). These inconsistencies may also reflect differences in study design, muscle selection, or population characteristics (34). In addition, age-related muscle fiber transitions—particularly type II fiber atrophy and Tc prolongation—have been implicated not only in sarcopenia but also in broader mobility decline associated with frailty, reinforcing the physiological relevance of TMG indicators in aging populations (34).

However, to our knowledge, there are currently no published studies that have directly applied TMG to clinically diagnosed sarcopenia populations. As mentioned earlier, Ziegl et al. (52) and Labata-Lezaun et al. (53) investigated the relationship between TMG parameters and physical performance in older adults, but their findings are limited by the absence of formally classified sarcopenic individuals. Furthermore, those older adults studied often represent a frailer subset within the aging population, with functional profiles that may partially overlap with sarcopenia despite lacking formal classification. Therefore, future studies should aim to include larger and clinically stratified samples to determine the diagnostic utility of TMG across sarcopenia phenotypes (34), such as the reference group with normal muscle mass and function, the functional deficit group, and the muscle loss group.

Importantly, our methodology minimized measurement variability by standardizing posture and joint angle during TMG assessments. All recordings were conducted in supine or prone positions depending on the anatomical location of each muscle, and foam supports were used to maintain consistent limb alignment. While some prior studies suggest that body position can influence TMG results, our protocol’s consistency supports the reliability of the measurements (54). Nevertheless, the absence of observable TMG differences in our sarcopenia groups highlights the need for further studies using larger samples, more diverse diagnostic criteria, or longitudinal designs to fully elucidate neuromechanical changes across disease stages (34). While TMG has demonstrated utility in differentiating neuromuscular impairments between PD and sarcopenia, the absence of significant TMG variation across sarcopenia classification groups in our study highlights the complexity of its application (26). These findings suggest that TMG sensitivity in detecting sarcopenia-related changes may depend on disease severity, muscle selection, and the comparison framework. Thus, TMG should be viewed as a complementary rather than standalone diagnostic approach for sarcopenia, particularly given its limited ability to assess voluntary neural activation and diagnostic specificity (27).

In practical terms, combining TMG with conventional strength measures could help clinicians identify whether a patient’s weakness originates from neural or structural factors. The present results indicate that TMG has the potential to differentiate central neuromuscular impairments associated with Parkinson’s disease from the peripheral muscular deficits observed in sarcopenia. This distinction is clinically relevant, as it provides a rationale for developing condition-specific rehabilitation strategies (52). This distinction is also supported by our statistical findings: TMG parameters were significantly impacted by PD status, which showed five significant main effects (on Td, Tc, Ts, Tr, and Vc) and one interaction effect (on Ts) across the six multivariate analyses. In contrast, sarcopenia status yielded no significant main effects for any TMG parameter.

This divergence implies that the pathological characteristics of PD have a more direct impact on contractile muscle properties, altering neuromuscular behavior via central mechanisms. Specifically, individuals with PD exhibited TMG abnormalities—such as prolonged Tc and reduced Dm—suggesting impaired neural activation and increased muscle stiffness. Conversely, individuals with sarcopenia exhibited generalized muscle weakness without corresponding changes in TMG variables, implying that their deficits are more closely associated with a structural or quantitative (mass-related) etiology rather than intrinsic changes in contractile timing or elasticity (20).

Although we emphasized the contribution of muscle mass to strength deficits, emerging evidence suggests that strength loss may actually precede muscle mass loss in some populations (55). For instance, a quantitative review by Mitchell et al. (55) demonstrated that muscle strength declines more rapidly and earlier than muscle mass with advancing age, highlighting the importance of neural impairment and muscle quality loss in early functional decline. Similarly, findings from longitudinal studies have shown that strength decline can precede detectable loss of muscle mass, further supporting the role of muscle quality and neural impairment in early functional decline (56–58).

Given that our TMG measurements targeted lower leg muscles (e.g., gastrocnemius, soleus), incorporating resistance training focused on these distal muscles may be particularly beneficial.

Recent studies have shown that lower-leg-targeted interventions—such as elastic band training (59) and high-intensity resistance exercises for the calf muscles can effectively improve muscle strength, mobility, and metabolic function in older adults. Therefore, resistance training programs encompassing both proximal and distal muscle groups may be more appropriate for mitigating sarcopenic weakness in diverse anatomical regions.

Third, the results of this study suggest that it is essential to consider functional status in addition to muscle mass when diagnosing and intervening in sarcopenia.

In the present study, classification based on sarcopenia-related criteria revealed that both reduced muscle mass and functional decline significantly impacted muscle strength.

Participants categorized into either the functional decline group or the reduced ASM (appendicular skeletal muscle mass) group exhibited decreased MVC across major muscle groups, with no statistically significant difference in strength observed between the two groups.

These findings emphasize that functional status is a critical factor in sarcopenia assessment and support the need for interventions targeting not only increases in muscle mass but also improvements in functional capacity.

Although no statistically significant interaction effects were observed between PD and sarcopenia, both conditions independently contributed to reductions in muscle strength.

Despite the absence of a significant interaction, the simultaneous presence of PD and sarcopenia should not be dismissed in clinical practice due to their distinct and potentially compounding effects.

PD impairs neuromuscular function through central motor dysfunction and altered muscle tone, whereas sarcopenia exerts its effects via peripheral muscle loss, metabolic decline, and structural deterioration (7, 20, 60, 61).

As a result, their coexistence may lead to an additive neuromechanical burden, manifesting as impaired postural control, mobility limitations, and increased fall risk (32).

For example, Lima et al. (31) found that approximately 60% of patients with PD with sarcopenia had experienced falls.

Furthermore, Liu et al. (62) and Ponsoni et al. (63) identified associations between sarcopenia and reduced functional ability, walking performance, and activities of daily living.

Moreover, Cai et al. (7) reported that 31.2% of patients with PD presented with sarcopenia, although no direct correlation was identified between the presence of sarcopenia and the severity of motor symptoms.

While these studies do not directly validate our findings, they reinforce the clinical importance of recognizing the risks associated with the co-occurrence of PD and sarcopenia (32).

Therefore, early identification and management of sarcopenia in patients with PD may play a key role in fall prevention and mobility preservation (31).

Fourth, neural mechanisms such as motor unit remodeling, impaired voluntary activation, and incomplete reinnervation likely contribute to strength loss in both conditions (30). Although not directly measured here, these elements play an important role in age-related neuromuscular decline (30, 64). This supports the concept that sarcopenia involves both central and peripheral pathophysiology. This perspective further supports that strength loss in sarcopenia is not solely attributable to muscle mass decline, but also reflects neural impairments contributing to functional deterioration (58, 65).

It is worth noting that all PD participants were assessed in their usual medication state, which may partly explain the variability observed in contractile responses. Although TMG parameters were measured under standardized postural and joint conditions—either supine or prone depending on the anatomical location of each muscle—other influencing factors, particularly medication use, were not statistically controlled, which may have influenced neuromuscular responses such as those captured by TMG. Major medications (e.g., levodopa, dopamine agonists) taken by the PD group were documented in Supplementary Table 1; however, due to inter-individual variability in dosage, timing, and pharmacodynamics, adjusting for medication effects was not feasible within our cross-sectional design (66). This remains a limitation of the present study. Future work should stratify PD participants by medication timing or dosage to disentangle dopaminergic modulation from neuromuscular performance.

Additional limitations should be acknowledged. First, the relatively small sample size may restrict the generalizability of findings. Second, the study focused on a limited number of lower limb muscles—mainly distal muscles—potentially overlooking the contributions of proximal or upper limb function. While both muscle function and mass were assessed in this study, we acknowledge that our cross-sectional design does not allow for inferences about longitudinal decline or changes over time. Third, while TMG provides useful mechanical data, it does not capture voluntary activation, motor unit recruitment, or central neural drive (15, 27). Non-significant TMG variations across sarcopenia classes likely indicate structural rather than neural changes, consistent with previous findings. Finally, as a cross-sectional study, causal inferences and temporal changes cannot be assessed. Future studies should include larger, more diverse populations and broader neuromuscular assessment tools to address these gaps. Although the sample size was modest, the consistent medium-to-large effect sizes observed across muscle parameters support the stability and reliability of our findings. Altogether, these insights add to the growing understanding that neural and structural mechanisms should be addressed together when managing mobility decline in aging and neurodegenerative conditions.

## 5 Conclusion

The current study suggests that PD and sarcopenia may independently affect muscle strength and contractile properties, delineating the functional limitations associated with each condition. Notably, PD significantly impacts specific muscle groups in the lower body, particularly extension and plantar flexion strength, potentially causing delayed muscle response and stiffness through increased Tc and decreased Dm. In contrast, sarcopenia appears to primarily contribute to a general decline in lower body strength and movement constraints through muscle mass reduction. Based on these condition-specific characteristics, future research should involve long-term studies tailored to each disease, focusing on not only muscle strength improvement but also enhancing muscle contractility and functionality to develop personalized rehabilitation and treatment programs. Although no intervention was included in the current study, the present findings provide a preliminary foundation for rehabilitation-oriented research.

## Data Availability

The original contributions presented in the study are included in the article/Supplementary material, further inquiries can be directed to the corresponding author.

## Supplementary material

Supplementary Figure 1 Representative postures for isometric force assessments in the lower limbs. Standardized postures for assessing isometric force in the hip, knee, and ankle joints. Images illustrate participant positioning and dynamometer placement for each task.

## References

[1] Rosin R Topka H Dichgans J. Gait initiation in Parkinson’s disease. *Mov Disord*. (1997) 12:682–90. 10.1002/mds.870120509 9380048

[2] Surmeier DJ Obeso JA Halliday GM. Selective neuronal vulnerability in Parkinson disease. *Nat Rev Neurosci*. (2017) 18:101–13. 10.1038/nrn.2016.178 28104909 PMC5564322

[3] Dauer W Przedborski S. Parkinson’s disease: mechanisms and models. *Neuron*. (2003) 39:889–909. 10.1016/s0896-6273(03)00568-3 12971891

[4] Falvo MJ Schilling BK Earhart GM. Parkinson’s disease and resistive exercise: rationale, review, and recommendations. *Mov Disord*. (2008) 23:1–11. 10.1002/mds.21690 17894327

[5] Kuopio AM Marttila RJ Helenius H Toivonen M Rinne UK. The quality of life in Parkinson’s disease. *Mov Disord.* (2000) 15:216–23. 10.1002/1531-8257(200003)15:2<216::aid-mds1003>3.0.co;2-#. 10752569

[6] Bloem BR Hausdorff JM Visser JE Giladi N. Falls and freezing of gait in Parkinson’s disease: a review of two interconnected, episodic phenomena. *Mov Disord*. (2004) 19:871–84. 10.1002/mds.20115 15300651

[7] Cai Y Feng F Wei Q Jiang Z Ou R Shang H. Sarcopenia in patients with Parkinson’s disease: a systematic review and meta-analysis. *Front Neurol*. (2021) 12:598035. 10.3389/fneur.2021.598035 33746871 PMC7973225

[8] World Health Organization [WHO] *Parkinson Disease.* Geneva: World Health Organization (2023).

[9] Su D Cui Y He C Yin P Bai R Zhu J et al. Projections for prevalence of Parkinson’s disease and its driving factors in 195 countries and territories to 2050: modelling study of Global Burden of Disease Study 2021. *BMJ*. (2021) 388:e080952. 10.1136/bmj-2024-080952 40044233 PMC11881235

[10] Durmus B Baysal O Altinayar S Altay Z Ersoy Y Ozcan C. Lower extremity isokinetic muscle strength in patients with Parkinson’s disease. *J Clin Neurosci*. (2010) 17:893–6. 10.1016/j.jocn.2009.11.014 20435478

[11] Karim A Iqbal MS Muhammad T Qaisar R. Evaluation of sarcopenia using biomarkers of the neuromuscular junction in Parkinson’s disease. *J Mol Neurosci*. (2022) 72:820–9. 10.1007/s12031-022-01970-7 35044622

[12] Rossi B Siciliano G Carboncini MC Manca ML Massetani R Viacava P et al. Muscle modifications in Parkinson’s disease: myoelectric manifestations. *Electroencephalogr Clin Neurophysiol*. (1996) 101:211–8. 10.1016/0924-980x(96)94672-x 8647033

[13] Kelly NA Hammond KG Bickel CS Windham ST Tuggle SC Bamman MM. Effects of aging and Parkinson’s disease on motor unit remodeling: influence of resistance exercise training. *J Appl Physiol*. (2018) 124:888–98. 10.1152/japplphysiol.00563.2017 29357501 PMC5972459

[14] Lavin KM Sealfon SC McDonald MN Roberts BM Wilk K Nair VD et al. Skeletal muscle transcriptional networks linked to type I myofiber grouping in Parkinson’s disease. *J Appl Physiol*. (2020) 128:229–40. 10.1152/japplphysiol.00702.2019 31829804 PMC7052589

[15] Simuniè B. Between-day reliability of a method for non-invasive estimation of muscle composition. *J Electromyogr Kinesiol*. (2012) 22:527–30. 10.1016/j.jelekin.2012.04.003 22546361

[16] Dodds RM Roberts HC Cooper C Sayer AA. The epidemiology of sarcopenia. *J Clin Densitom*. (2015) 18:461–6. 10.1016/j.jocd.2015.04.012 26073423 PMC4629409

[17] Shafiee G Keshtkar A Soltani A Ahadi Z Larijani B Heshmat R. Prevalence of sarcopenia in the world: a systematic review and meta- analysis of general population studies. *J Diabetes Metab Disord*. (2017) 16:1–10. 10.1186/s40200-017-0302-x 28523252 PMC5434551

[18] Herz DM Brown P. Moving, fast and slow: behavioural insights into bradykinesia in Parkinson’s disease. *Brain*. (2023) 146:3576–86. 10.1093/brain/awad069 36864683 PMC10473574

[19] van der Kolk NM King LA. Effects of exercise on mobility in people with Parkinson’s disease. *Mov Disord*. (2013) 28:1587–96. 10.1002/mds.25658 24132847

[20] Cruz-Jentoft AJ Bahat G Bauer J Boirie Y Bruyère O Cederholm T et al. Sarcopenia: revised European consensus on definition and diagnosis. *Age Ageing*. (2019) 48:16–31. 10.1093/ageing/afy169 30312372 PMC6322506

[21] Bhasin S Travison TG Manini TM Patel S Pencina KM Fielding RA et al. Sarcopenia definition: the position statements of the sarcopenia definition and outcomes consortium. *J Am Geriatr Soc*. (2020) 68:1410–8. 10.1111/jgs.16372 32150289 PMC12132920

[22] Chen LK Woo J Assantachai P Auyeung TW Chou MY Iijima K et al. Asian Working Group for Sarcopenia: 2019 consensus update on sarcopenia diagnosis and treatment. *J Am Med Dir Assoc.* (2020) 21:300–307.e2. 10.1016/j.jamda.2019.12.012. 32033882

[23] Landers KA Hunter GR Wetzstein CJ Bamman MM Weinsier RL. The interrelationship among muscle mass, strength, and the ability to perform physical tasks of daily living in younger and older women. *J Gerontol A Biol Sci Med Sci*. (2001) 56:B443–8. 10.1093/gerona/56.10.b443 11584029

[24] Narici MV Maffulli N. Sarcopenia: characteristics, mechanisms and functional significance. *Br Med Bull*. (2010) 95:139–59. 10.1093/bmb/ldq008 20200012

[25] Dahmane R Valen IV Knez N Er en I. Evaluation of the ability to make non-invasive estimation of muscle contractile properties on the basis of the muscle belly response. *Med Biol Eng Comput*. (2001) 39:51–5. 10.1007/BF02345266 11214273

[26] Simunič B Degens H Rittweger J Narici M Mekjavić IB Pišot R. Noninvasive estimation of myosin heavy chain composition in human skeletal muscle. *Med Sci Sports Exerc*. (2011) 43:1619–25. 10.1249/MSS.0b013e31821522d0 21552151

[27] Macgregor LJ Hunter AM Orizio C Fairweather MM Ditroilo M. Assessment of skeletal muscle contractile properties by radial displacement: the case for tensiomyography. *Sports Med*. (2018) 48:1607–20. 10.1007/s40279-018-0912-6 29605838 PMC5999145

[28] Gamborg M Hvid LG Thrue C Johansson S Franzén E Dalgas U et al. Muscle strength and power in people with Parkinson disease: a systematic review and meta-analysis. *J Neurol Phys Ther*. (2023) 47:3–15. 10.1097/NPT.0000000000000421 36318503

[29] Schlenstedt C Paschen S Kruse A Raethjen J Weisser B Deuschl G. Resistance versus balance training to improve postural control in parkinson’s disease: a randomized rater blinded controlled study. *PLoS One*. (2015) 10:e0140584. 10.1371/journal.pone.0140584 26501562 PMC4621054

[30] Hunter SK Pereira HM Keenan KG. The aging neuromuscular system and motor performance. *J Appl Physiol*. (2016) 121:982–95. 10.1152/japplphysiol.00475.2016 27516536 PMC5142309

[31] Lima DP de Almeida SB Bonfadini JC de Luna JRG de Alencar MS Pinheiro-Neto EB et al. Clinical correlates of sarcopenia and falls in Parkinson’s disease. *PLoS One*. (2020) 15:e0227238. 10.1371/journal.pone.0227238 32191713 PMC7082018

[32] Hart A Cordova-Rivera L Barker F Sayer AA Granic A Yarnall AJ. The prevalence of sarcopenia in Parkinson’s disease and related disorders- a systematic review. *Neurol Sci*. (2023) 44:4205–17. 10.1007/s10072-023-07007-0 37594550 PMC10641055

[33] Koo BK. Assessment of muscle quantity, quality and function. *J Obes Metab Syndr*. (2022) 31:9–16. 10.7570/jomes22025 35318289 PMC8987447

[34] Pus K Paravlic AH Šimuniè B. The use of tensiomyography in older adults: a systematic review. *Front Physiol*. (2023) 14:1213993. 10.3389/fphys.2023.1213993 37398907 PMC10311920

[35] Kehagia AA North TK Grose J Jeffery AN Cocking L Chapman R et al. Enhancing trial delivery in Parkinson’s Disease: qualitative insights from PD STAT. *J Parkinsons Dis*. (2022) 12:1591–604. 10.3233/JPD-212987 35466952 PMC9398073

[36] Tarolli CG. Author response: symptom burden among individuals with Parkinson disease: a national survey. *Neurol Clin Pract*. (2020) 10:90. 10.1212/CPJ.0000000000000817 32309022 PMC7156199

[37] Button KS Ioannidis JP Mokrysz C Nosek BA Flint J Robinson ES et al. Power failure: why small sample size undermines the reliability of neuroscience. *Nat Rev Neurosci*. (2013) 14:365–76. 10.1038/nrn3475 23571845

[38] Schlesselman JJ. *Case-Control Studies: Design, Conduct, Analysis.* Oxford: Oxford university press (1982).

[39] Goetz CG Poewe W Rascol O Sampaio C Stebbins GT Counsell C et al. Movement disorder society task force report on the Hoehn and Yahr staging scale: status and recommendations. *Mov Disord*. (2004) 19:1020–8. 10.1002/mds.20213 15372591

[40] Oshima Y Shiga T Namba H Kuno S. Estimation of whole-body skeletal muscle mass by bioelectrical impedance analysis in the standing position. *Obes Res Clin Pract*. (2010) 4:e1–82. 10.1016/j.orcp.2009.06.001 24345620

[41] Greene BR McGrath D O’Neill R O’Donovan KJ Burns A Caulfield B. An adaptive gyroscope-based algorithm for temporal gait analysis. *Med Biol Eng Comput*. (2010) 48:1251–60. 10.1007/s11517-010-0692-0 21042951

[42] Tous-Fajardo J Moras G Rodríguez-Jiménez S Usach R Doutres DM Maffiuletti NA. Inter-rater reliability of muscle contractile property measurements using non-invasive tensiomyography. *J Electromyogr Kinesiol*. (2010) 20:761–6. 10.1016/j.jelekin.2010.02.008 20236839

[43] Langen G Lohr C Ueberschär O Behringer M. Reproducibility of knee extensor and flexor contraction velocity in healthy men and women assessed using tensiomyography: a registered report. *PLoS One*. (2023) 18:e0288806. 10.1371/journal.pone.0288806 37531344 PMC10395843

[44] Neptune RR Clark DJ Kautz SA. Modular control of human walking: a simulation study. *J Biomech*. (2009) 42:1282–7. 10.1016/j.jbiomech.2009.03.009 19394023 PMC2696580

[45] Park TS Shin MJ. Comprehensive assessment of lower limb function and muscle strength in Sarcopenia: insights from the Sit-to-Stand Test. *Ann Geriatr Med Res*. (2024) 28:1–8. 10.4235/agmr.23.0205 38325818 PMC10982452

[46] Li X He J Yun J Qin H. Lower limb resistance training in individuals with Parkinson’s disease: an updated systematic review and meta-analysis of randomized controlled trials. *Front Neurol*. (2020) 11:591605. 10.3389/fneur.2020.591605 33281732 PMC7691593

[47] Mateos-Toset S Cabrera-Martos I Torres-Sánchez I Ortiz-Rubio A González-Jiménez E Valenza MC. Effects of a single hand-exercise session on manual dexterity and strength in persons with Parkinson Disease: a randomized controlled trial. *PM R*. (2016) 8:115–22. 10.1016/j.pmrj.2015.06.004 26079867

[48] Beaudart C Demonceau C Reginster JY Locquet M Cesari M Cruz Jentoft AJ et al. Sarcopenia and health-related quality of life: a systematic review and meta-analysis. *J Cachexia Sarcopenia Muscle*. (2023) 14:1228–43. 10.1002/jcsm.13243 37139947 PMC10235892

[49] Inkster LM Eng JJ MacIntyre DL Stoessl AJ. Leg muscle strength is reduced in Parkinson’s disease and relates to the ability to rise from a chair. *Mov Disord*. (2003) 18:157–62. 10.1002/mds.10299 12539208 PMC3471985

[50] David FJ Robichaud JA Vaillancourt DE Poon C Kohrt WM Comella CL et al. Progressive resistance exercise restores some properties of the triphasic EMG pattern and improves bradykinesia: the PRET-PD randomized clinical trial. *J Neurophysiol*. (2016) 116:2298–311. 10.1152/jn.01067.2015 27582297 PMC5110637

[51] Allen NE Sherrington C Canning CG Fung VS. Reduced muscle power is associated with slower walking velocity and falls in people with Parkinson’s disease. *Parkinsonism Relat Disord*. (2010) 16:261–4. 10.1016/j.parkreldis.2009.12.011 20117036

[52] Ziegl A Hayn D Kastner P Fabiani E Šimunič B Löffler K et al. Quantification of the link between timed up-and-go test subtasks and contractile muscle properties. *Sensors*. (2021) 21:6539. 10.3390/s21196539 34640875 PMC8512551

[53] Labata-Lezaun N González-Rueda V Llurda-Almuzara L López-de-Celis C Rodríguez-Sanz J Cadellans-Arróniz A et al. Correlation between physical performance and tensiomyographic and myotonometric parameters in older adults. *Healthcare*. (2023) 11:2169. 10.3390/healthcare11152169 37570409 PMC10418601

[54] Pajović L Toskić L Stanković V Lilić L Cicović B. Muscle contractile properties measured by the Tensiomyography (TMG) method in top-level football players of different playing positions: the case of serbian super league. *Int J Environ Res Public Health*. (2023) 20:924. 10.3390/ijerph20020924 36673686 PMC9859018

[55] Mitchell WK Williams J Atherton P Larvin M Lund J Narici M. Sarcopenia, dynapenia, and the impact of advancing age on human skeletal muscle size and strength; a quantitative review. *Front Physiol*. (2012) 3:260. 10.3389/fphys.2012.00260 22934016 PMC3429036

[56] Lindle RS Metter EJ Lynch NA Fleg JL Fozard JL Tobin J et al. Age and gender comparisons of muscle strength in 654 women and men aged 20-93 yr. *J Appl Physiol*. (1997) 83:1581–7. 10.1152/jappl.1997.83.5.1581 9375323

[57] Hughes VA Frontera WR Wood M Evans WJ Dallal GE Roubenoff R et al. Longitudinal muscle strength changes in older adults: influence of muscle mass, physical activity, and health. *J Gerontol A Biol Sci Med Sci*. (2001) 56:B209–17. 10.1093/gerona/56.5.b209 11320101

[58] Delmonico MJ Harris TB Visser M Park SW Conroy MB Velasquez-Mieyer P et al. Longitudinal study of muscle strength, quality, and adipose tissue infiltration. *Am J Clin Nutr*. (2009) 90:1579–85. 10.3945/ajcn.2009.28047 19864405 PMC2777469

[59] Seo MW Jung SW Kim SW Lee JM Jung HC Song JK. Effects of 16 weeks of resistance training on muscle quality and muscle growth factors in older adult women with sarcopenia: a randomized controlled trial. *Int J Environ Res Public Health*. (2021) 18:6762. 10.3390/ijerph18136762 34201810 PMC8267934

[60] Landi F Calvani R Picca A Tosato M Martone AM Ortolani E et al. Cardiovascular health metrics, muscle mass and function among Italian community-dwellers: the Lookup 7+ project. *Eur J Public Health*. (2019) 28:766–72. 10.1093/eurpub/cky034 29554257

[61] Frazzitta G Bertotti G Riboldazzi G Turla M Uccellini D Boveri N et al. Effectiveness of intensive inpatient rehabilitation treatment on disease progression in parkinsonian patients: a randomized controlled trial with 1-year follow-up. *Neurorehabil Neural Repair*. (2012) 26:144–50. 10.1177/1545968311416990 21844282

[62] Liu G Hong C Xu S Huang Y Zheng F Gao Y et al. Association of sarcopenia with Parkinson’s disease and related functional degeneration among older adults: a prospective cohort study in Europe. *J Affect Disord*. (2025) 374:553–62. 10.1016/j.jad.2025.01.084 39837464

[63] Ponsoni A Sardeli AV Costa FP Mourão LF. Prevalence of sarcopenia in Parkinson’s disease: a systematic review and meta-analysis. *Geriatr Nurs*. (2023) 49:44–9. 10.1016/j.gerinurse.2022.11.006 36413812

[64] Nùñez-Lisboa M Valero-Breton M Dewolf AH. Unraveling age-related impairment of the neuromuscular system: exploring biomechanical and neurophysiological perspectives. *Front Physiol*. (2023) 14:1194889. 10.3389/fphys.2023.1194889 37427405 PMC10323685

[65] Clark BC Manini TM. What is dynapenia? *Nutrition*. (2012) 28:495–503. 10.1016/j.nut.2011.12.002 22469110 PMC3571692

[66] Tomlinson CL Stowe R Patel S Rick C Gray R Clarke CE. Systematic review of levodopa dose equivalency reporting in Parkinson’s disease. *Mov Disord*. (2010) 25:2649–53. 10.1002/mds.23429 21069833

