# Supplementary material for: Cross-sectional comparison of lower-limb muscle strength and contractile properties according to Parkinson’s disease and sarcopenia status
Source: Front Med (Lausanne). 2026 Mar 20;13:1546672. doi: 10.3389/fmed.2026.1546672 (PMC13047914; doi:10.3389/fmed.2026.1546672)
Supplement: Supplementary file 1 [file Table_1.docx]

# Parkinson’s Disease Patient Summary (with INN Medications)

| subject ID | Disease duration (based on diagnosis) | Hoehn-Yahr scale | Motor scale | ADL | UPDRS | Medication |
| --- | --- | --- | --- | --- | --- | --- |
| 1 | 1.8 | 1.0 | 7 | 10 |  | Levodopa/Carbidopa/Entacapone, Pramipexole 0.375mg, Acetylcysteine, Trihexyphenidyl |
| 2 | 2.3 | 2.0 | 5 | 1 |  | Levodopa/Carbidopa/Entacapone, Pramipexole 0.5mg, Rasagiline |
| 3 | 3.0 | 2.0 | 8 | 7 | 3.0 | Levodopa/Carbidopa, Pramipexole 0.625mg, Selegiline |
| 4 | 1.25 | 1.0 | 1 | 2 |  | Levodopa/Carbidopa, Levodopa/Carbidopa/Entacapone, Pramipexole 0.25mg, Selegiline, Amantadine |
| 5 | 4.0 | 2.5 | 17 | 7 |  | Levodopa/Carbidopa, Pramipexole 0.5mg, Zolpidem 5mg, Alprazolam 0.25mg |
| 6 | 5.5 | 2.5 | 41 | 13 |  | Pramipexole 0.25mg, Rasagiline |
| 7 | 6.2 | 3.0 | 23 | 14 | 12.0 | Levodopa/Benserazide, Levodopa/Carbidopa/Entacapone, Pramipexole 0.125mg, Midodrine, Choline Alfoscerate |
| 8 | 5.0 | 2.0 | 5 | 3 | 3.0 | Pramipexole ER, Levodopa/Carbidopa, Levodopa/Carbidopa/Entacapone, Pramipexole, Mecobalamin, Trazodone, Alprazolam 0.25mg, Lorazepam |
| 9 | 3.6 | 1.0 | 25 | 13 |  | Levodopa/Carbidopa/Entacapone, Pramipexole 0.375mg, Pramipexole ER 0.375mg, Rasagiline |
| 10 | 6.8 | 2.0 | 29 | 12 |  | Midodrine, Fludrocortisone, Levodopa/Benserazide, Levodopa/Carbidopa/Entacapone, Pramipexole 0.5mg, Rasagiline, Aspirin, Mecobalamin, Zolpidem, Triamterene, Alprazolam |
| 11 | 4.9 | 2.0 | 16 | 4 | 5.0 | Levodopa/Carbidopa, Levodopa/Carbidopa/Entacapone, Pramipexole 0.25mg, Choline Alfoscerate, Pramipexole ER 0.375mg, Amantadine, Selegiline |
| 12 | 4.2 | 2.0 | 18 | 6 |  | Levodopa/Carbidopa, Pramipexole 0.375mg, Levodopa/Carbidopa/Entacapone, Rasagiline |
| 13 | 4.75 | 2.5 | 22 | 8 |  | Levodopa/Carbidopa, Pramipexole 0.5mg, Selegiline, Alprazolam 0.25mg |
| 14 | 2.0 | 1.0 | 4 | 2 | 1.0 | Levodopa/Carbidopa/Entacapone, Pramipexole 0.5mg, Clonazepam 0.5mg |
| 15 | 2.5 | 1.0 | 11 | 5 | 0.0 | Pramipexole 0.375mg, Levodopa/Carbidopa, Levodopa/Carbidopa/Entacapone |
| 16 | 5.4 | 1.0 | 37 | 14 | 9.0 | Levodopa/Carbidopa/Entacapone, Pramipexole 1mg, Selegiline, Alprazolam 0.25mg, Diazepam 2mg |
| 17 | 9.3 | 2.5 | 63 | 35 |  | Levodopa/Carbidopa, Levodopa/Carbidopa/Entacapone, Pramipexole 0.375mg, Droxidopa, Selegiline |
| 18 | 3.25 | 2.5 | 12 | 8 |  | No medication |
| 19 | 2.75 | 2.0 | 7 | 2 | 4.0 | Levodopa/Carbidopa, Levodopa/Carbidopa/Entacapone, Pramipexole 0.5mg, Rasagiline |
| 20 | 1.5 | 1.0 | 4 | 2 | 2.0 | Levodopa/Carbidopa/Entacapone (75/18.75/200mg), Pramipexole 0.25mg |
