# Supplementary material for: Cross-sectional comparison of lower-limb muscle strength and contractile properties according to Parkinson’s disease and sarcopenia status
Source: Front Med (Lausanne). 2026 Mar 20;13:1546672. doi: 10.3389/fmed.2026.1546672 (PMC13047914; doi:10.3389/fmed.2026.1546672)
Supplement: Supplementary file 2 [file Table_2.docx]

# Supplementary Table 2. Standardized Isometric Force Assessment Postures for Lower Limb Tasks

| Panel | Movement | Participant Position | Dynamometer Placement | Instruction to Participant |
| --- | --- | --- | --- | --- |
| A | Hip flexion | Seated upright; hip and knee at 90° flexion | Anterior distal thigh, just above the knee | Push upward with the thigh against the resistance without moving the hip or knee |
| B | Hip extension | Prone position; hip neutral, knee flexed to 90° | Posterior distal thigh | Extend the hip against the resistance while maintaining pelvic stability |
| C | Hip abduction | Side-lying; test leg extended upward, bottom leg flexed | Lateral distal thigh, proximal to knee | Lift the top leg directly upward without hip rotation against the resistance |
| D | Knee flexion | Prone position; hip neutral, knee flexed to 90° | Posterior lower leg, just above ankle | Flex the knee by pulling the heel toward the buttocks against the resistance |
| E | Knee extension | Seated upright; hip and knee at 90° flexion | Anterior lower leg, just above ankle | Extend the knee forward against the resistance, keeping the thigh stable |
| F | Ankle plantarflexion | Supine position; knee extended, ankle neutral | Dorsal aspect of forefoot | Press the ball of the foot downward as if performing a toe press |
| G | Ankle dorsiflexion | Supine position; knee extended, ankle neutral | Plantar surface of forefoot | Pull the toes and foot upward against the resistance |
