# Supplementary material for: Cross-sectional comparison of lower-limb muscle strength and contractile properties according to Parkinson’s disease and sarcopenia status
Source: Front Med (Lausanne). 2026 Mar 20;13:1546672. doi: 10.3389/fmed.2026.1546672 (PMC13047914; doi:10.3389/fmed.2026.1546672)
Supplement: Supplementary file 3 [file Table_3.docx]

# Supplementary Table 3. Full Ranked MANCOVA Outputs (Force and TMG)

This table presents the complete ranked MANCOVA results for isometric muscle strength (MVC Force) and TMG-derived parameters (Td, Tc, Ts, Tr, Dm, Vc). The table includes Wilks’ Lambda values, degrees of freedom (df), F-statistics, exact p-values, and partial eta-squared (η²) for the main effects of Parkinson’s disease (PD), sarcopenia status, and their interaction.

| Parameter | Effect | Wilks’ Lambda | df | F | p-value | Partial η² |
| --- | --- | --- | --- | --- | --- | --- |
| MVC Force | PD | .598 | 9,45 | 3.36 | .003 | .402 |
| MVC Force | Sarcopenia | .430 | 18,90 | 2.63 | .001 | .344 |
| MVC Force | PD × Sarcopenia | .691 | 18,90 | 1.01 | .453 | .169 |
| Td | PD | .706 | 4,50 | 5.21 | .001 | .294 |
| Td | Sarcopenia | .875 | 8,100 | 1.49 | .171 | .106 |
| Td | PD × Sarcopenia | .881 | 8,100 | 1.29 | .257 | .094 |
| Tc | PD | .638 | 4,50 | 7.10 | <.001 | .362 |
| Tc | Sarcopenia | .962 | 8,100 | 0.69 | .700 | .052 |
| Tc | PD × Sarcopenia | .964 | 8,100 | 0.65 | .730 | .050 |
| Ts | PD | .806 | 4,50 | 3.01 | .027 | .194 |
| Ts | Sarcopenia | .983 | 8,100 | 0.22 | .987 | .017 |
| Ts | PD × Sarcopenia | .719 | 8,100 | 2.24 | .031 | .152 |
| Tr | PD | .755 | 4,50 | 4.05 | .006 | .245 |
| Tr | Sarcopenia | .954 | 8,100 | 0.46 | .880 | .036 |
| Tr | PD × Sarcopenia | .940 | 8,100 | 0.82 | .585 | .062 |
| Dm | PD | .879 | 4,50 | 2.15 | .088 | .147 |
| Dm | Sarcopenia | .950 | 8,100 | 0.81 | .599 | .061 |
| Dm | PD × Sarcopenia | .938 | 8,100 | 0.83 | .576 | .062 |
| Vc | PD | .769 | 4,50 | 3.75 | .010 | .231 |
| Vc | Sarcopenia | .909 | 8,100 | 1.24 | .282 | .091 |
| Vc | PD × Sarcopenia | .911 | 8,100 | 1.22 | .295 | .089 |
