# Supplementary material for: Cross-sectional comparison of lower-limb muscle strength and contractile properties according to Parkinson’s disease and sarcopenia status
Source: Front Med (Lausanne). 2026 Mar 20;13:1546672. doi: 10.3389/fmed.2026.1546672 (PMC13047914; doi:10.3389/fmed.2026.1546672)
Supplement: Supplementary file 4 [file Table_4.docx]

# Supplementary Table 4. Post Hoc Ranked ANCOVA – Isometric Force Outputs

This table presents the results of post hoc ranked ANCOVA analyses for isometric force outputs where significant multivariate effects were detected. Each entry includes the F-statistic, exact p-value, and partial eta-squared (η²) for each comparison.

| Isometric Force Variable | F | p-value | η² |
| --- | --- | --- | --- |
| Hip Abduction | F(1,50) = 4.72 | .035 | .086 |
| Ankle Plantar Flexion | F(1,50) = 9.87 | .003 | .165 |
| Knee Extension (↑ in PD) | F(1,50) = 4.31 | .043 | .079 |
| Grip Strength | F(2,50) = 10.83 | <.001 | .302 |
| Pinch Strength | F(2,50) = 5.29 | .008 | .175 |
| Hip Extension | F(2,50) = 7.41 | .002 | .244 |
| Hip Flexion | F(2,50) = 6.14 | .004 | .215 |
| Knee Extension (sarc) | F(2,50) = 5.01 | .010 | .191 |
| Ankle Dorsiflexion | F(2,50) = 8.63 | .001 | .257 |
