# Supplementary material for: Cross-sectional comparison of lower-limb muscle strength and contractile properties according to Parkinson’s disease and sarcopenia status
Source: Front Med (Lausanne). 2026 Mar 20;13:1546672. doi: 10.3389/fmed.2026.1546672 (PMC13047914; doi:10.3389/fmed.2026.1546672)
Supplement: Supplementary file 5 [file Table_5.docx]

# Supplementary Table 5. Pairwise Comparisons – Isometric Force Outputs

Pairwise comparisons of isometric force outputs between groups based on PD status and sarcopenia status. Values include mean differences, 95% confidence intervals (CI), and Bonferroni-adjusted p-values.

| Muscle | Group Comparison | Mean Difference | 95% CI (Lower) | 95% CI (Upper) | p (Bonferroni) |
| --- | --- | --- | --- | --- | --- |
| Hip Flexion | PD vs. non-PD | -6.5 | -12.3 | -0.7 | 0.028 |
| Hip Extension | PD vs. non-PD | -4.2 | -10.1 | 1.7 | 0.172 |
| Hip Flexion | Muscle loss vs. Non-sarcopenia | -8.9 | -15.2 | -2.6 | 0.007 |
| Knee Extension | Functional decline vs. Non-sarcopenia | -5.7 | -11.0 | -0.4 | 0.035 |
| Ankle Dorsiflexion | Muscle loss vs. Functional decline | -3.3 | -8.5 | 1.9 | 0.243 |
