# Supplementary material for: Cross-sectional comparison of lower-limb muscle strength and contractile properties according to Parkinson’s disease and sarcopenia status
Source: Front Med (Lausanne). 2026 Mar 20;13:1546672. doi: 10.3389/fmed.2026.1546672 (PMC13047914; doi:10.3389/fmed.2026.1546672)
Supplement: Supplementary file 6 [file Table_6.docx]

# Supplementary Table 6. Pairwise Comparisons – TMG Parameters

This table presents pairwise comparisons of TMG parameters that showed significant differences, primarily focusing on the PD vs. non-PD group. Parameters include Tr and Dm. Sarcopenia-related comparisons were not significant and are not included. The results include mean differences, 95% confidence intervals, and Bonferroni-adjusted p-values.

| TMG Parameter | Comparison Group | Mean Difference | 95% CI | Bonferroni-adjusted p |
| --- | --- | --- | --- | --- |
| Tr (SO) | PD vs. non-PD | 16.53 | [5.20, 27.86] | 0.005 |
| Dm (GL) | PD vs. non-PD | 13.20 | [1.45, 24.94] | 0.028 |
| Dm (SO) | PD vs. non-PD | 13.65 | [1.85, 25.45] | 0.024 |
