# Supplementary figures and images for: Cross-sectional comparison of lower-limb muscle strength and contractile properties according to Parkinson’s disease and sarcopenia status
Source: Front Med (Lausanne). 2026 Mar 20;13:1546672. doi: 10.3389/fmed.2026.1546672 (PMC13047914; doi:10.3389/fmed.2026.1546672)

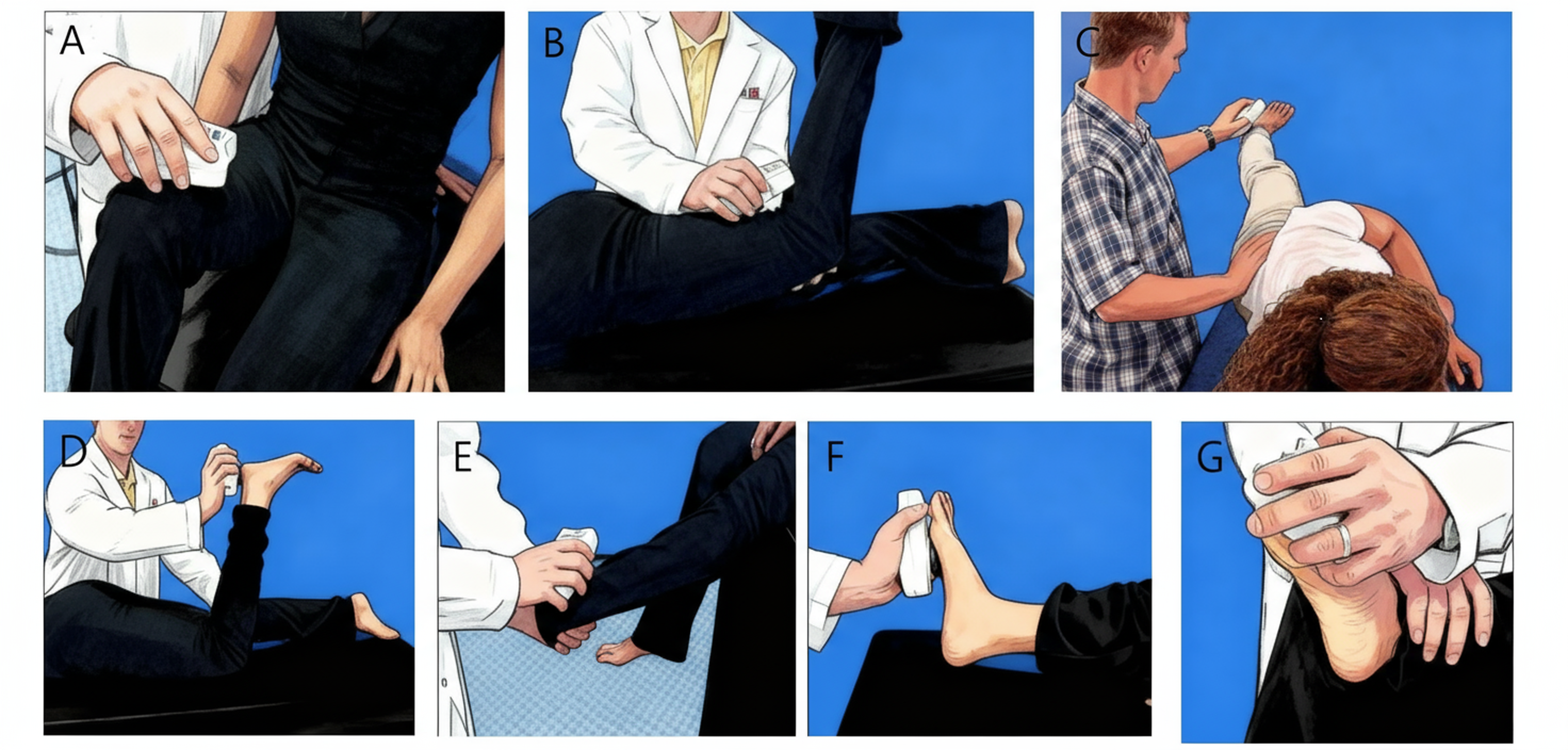

Supplement: Supplementary Figure 1 — Representative postures for isometric force assessments in the lower limbs. Standardized postures for assessing isometric force in the hip, knee, and ankle joints. Images illustrate participant positioning and dynamometer placement for each task. [file Image_1.tiff]
